# Intramolecular London Dispersion Interactions in Single-Molecule Junctions

**DOI:** 10.1021/jacs.3c12183

**Published:** 2024-02-07

**Authors:** Matthew O. Hight, Joshua Y. Wong, Ashley E. Pimentel, Timothy A. Su

**Affiliations:** † Department of Chemistry, University of California, Riverside, California 92521, United States; ‡ Materials Science & Engineering Program, University of California, Riverside, California 92521, United States

## Abstract

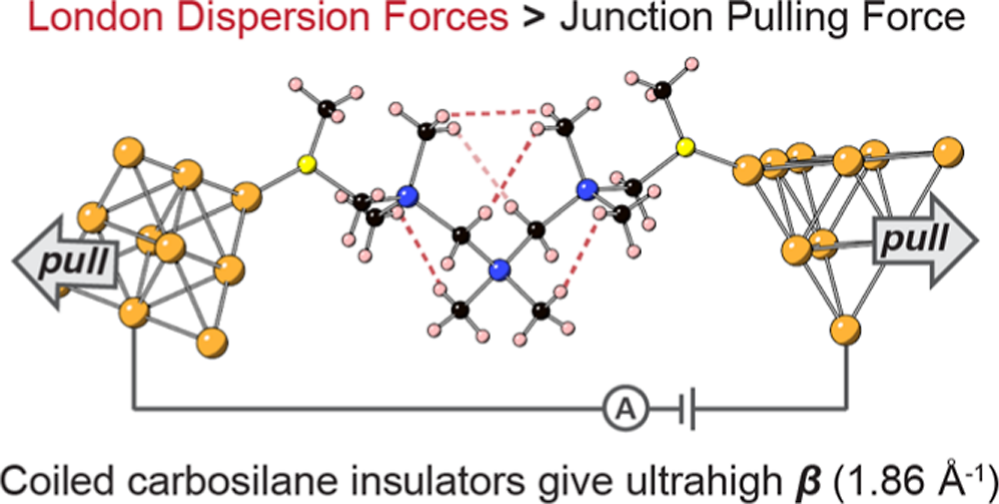

This work shows the first example of using intramolecular London dispersion interactions to control molecular geometry and quantum transport in single-molecule junctions. Flexible σ-bonded molecular junctions typically occupy straight-chain geometries due to steric effects. Here, we synthesize a series of thiomethyl-terminated oligo(dimethylsilmethylene)s that bear [CH_2_–Si(CH_3_)_2_]_*n*_ repeat units, where all backbone dihedral states are sterically equivalent. Scanning tunneling microscopy break-junction (STM-BJ) measurements and theoretical calculations indicate that in the absence of a strong steric bias concerted intramolecular London dispersion interactions staple the carbosilane backbone into coiled conformations that remain intact even as the junction is stretched to its breakpoint. As these kinked conformations are highly resistive to electronic transport, we observe record-high conductance decay values on an experimental junction length basis (β = 1.86 ± 0.12 Å^–1^). These studies reveal the potential of using intramolecular London dispersion interactions to design single-molecule electronics.

## Introduction

In isolation, a single London dispersion interaction is the weakest noncovalent interaction. However, when many concurrent London dispersion attractions occur simultaneously within a molecule, they can offer pronounced stabilization of sterically crowded molecular conformations that would otherwise be considered unfavorable.^1,2^ A groundswell of work exploiting this phenomenon has recently emerged across chemical disciplines. Intramolecular London dispersion forces are now deliberately built into catalyst design to improve the reaction rate and selectivity^3−10^ and into photoswitch design to control the thermal reaction rate and isomer stability.^11−14^ This article describes the first example of using intramolecular London dispersion interactions to control charge transport in molecular electronics.

The motivation for our work lies in the broader interest of the single-molecule electronics community to design and use individual molecules as the active components (e.g., insulators, wires, rectifiers, switches) in electronic circuitry.^15^ A key strategy for controlling electronic transport in a single-molecule junction is to control its conformation, since conformation dictates the strength of conjugation or coupling between electrodes through the molecular backbone.^16^ One approach to achieve this conformational control is to incorporate intramolecular interactions such as π-stacking and hydrogen bonding into the single-molecule backbone.^17−25^ Yang, and co-workers recently described London dispersion σ-interactions to control the association between molecules in stacked supramolecular junctions;^26^ yet it remains an open question as to whether these interactions are strong enough to control backbone geometry in single-molecule junctions. Until this point, London dispersion forces have not been observed in single-molecule measurements.

**Figure 1:**
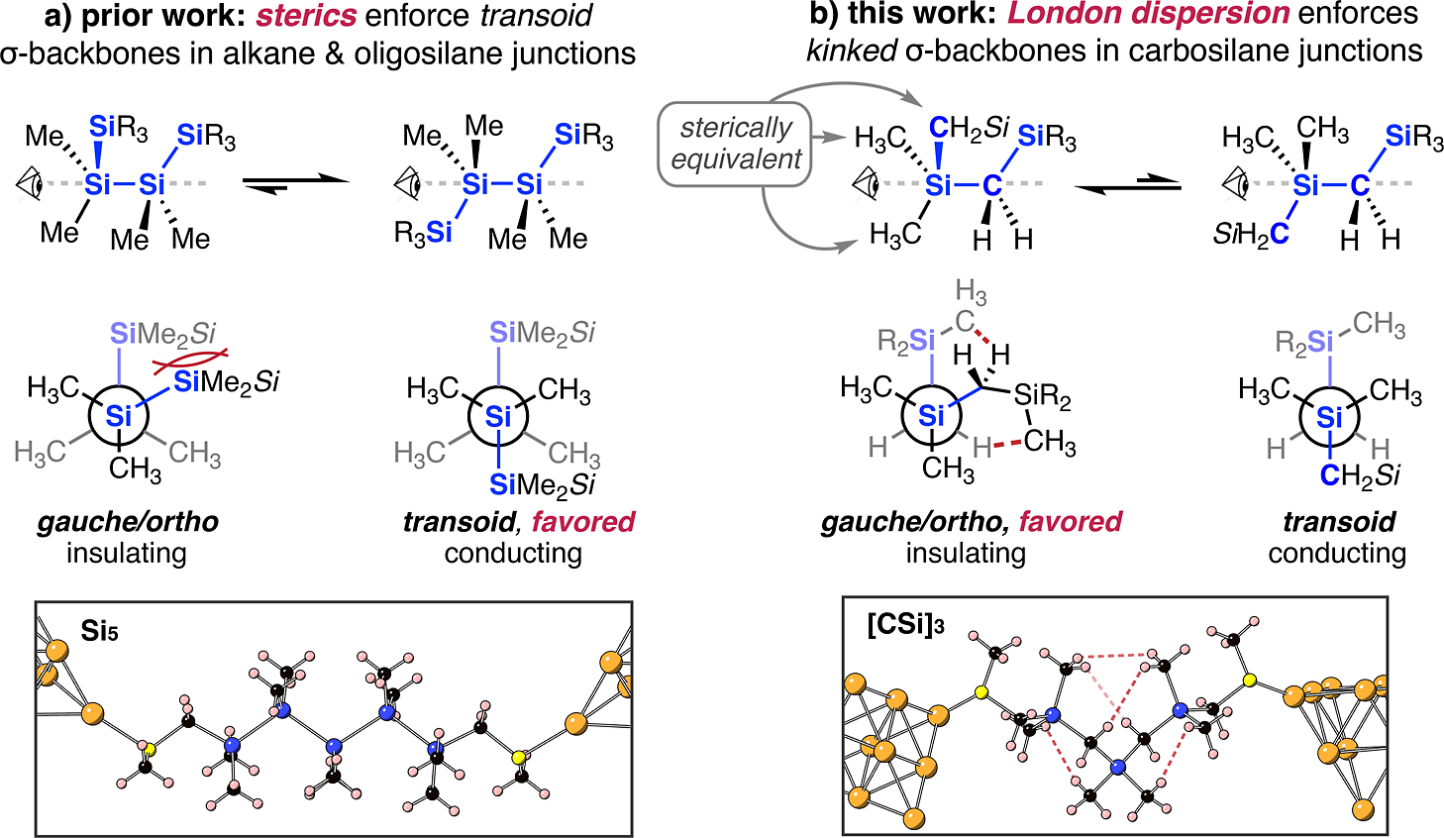
(a) Prior work in σ-bonded tetrel (CH_2_, SiMe_2_, or GeMe_2_) backbones favor anti (ω = 180°, alkanes) or transoid (ω = ± 165°, oligosilanes) conformers that minimize the steric repulsion (red lines) occurring in kinked dihedral geometries. (b) Methyl and silmethylene groups are sterically equivalent in PDMSM backbones. Under these circumstances, stabilizing intramolecular London dispersion interactions (dotted red lines) dictate backbone conformation, favoring coiled yet highly insulating single-molecule junction geometries. The junction structures of **Si**_**5**_ and **[CSi]**_**3**_ are colored as follows: Au (gold), S (yellow), C (black), Si (blue), and H (pink).

This gap in the literature can be ascribed to the fact that most single-molecule wires with flexible σ-backbones have conformations that are overwhelmingly influenced by steric effects (Figure 1a). Though alkane wires are freely rotating, their backbones predominately exist in anti (ω = 180°) dihedral geometries due to repulsive steric crowding in their gauche (ω = 60°) conformations. The same is true in permethylated oligosilane and oligogermane wires,^27−29^ where transoid (ω ∼ 165°) geometries are favored over gauche (ω ∼ 55°) and ortho (ω ∼ 90°) ones due to steric arguments (Figure 1a).^30^ These anti or transoid geometries become even more favored upon junction elongation, since the mechanical stretching axis is colinear with the straight-chain σ-bond axis.

**Figure 2:**
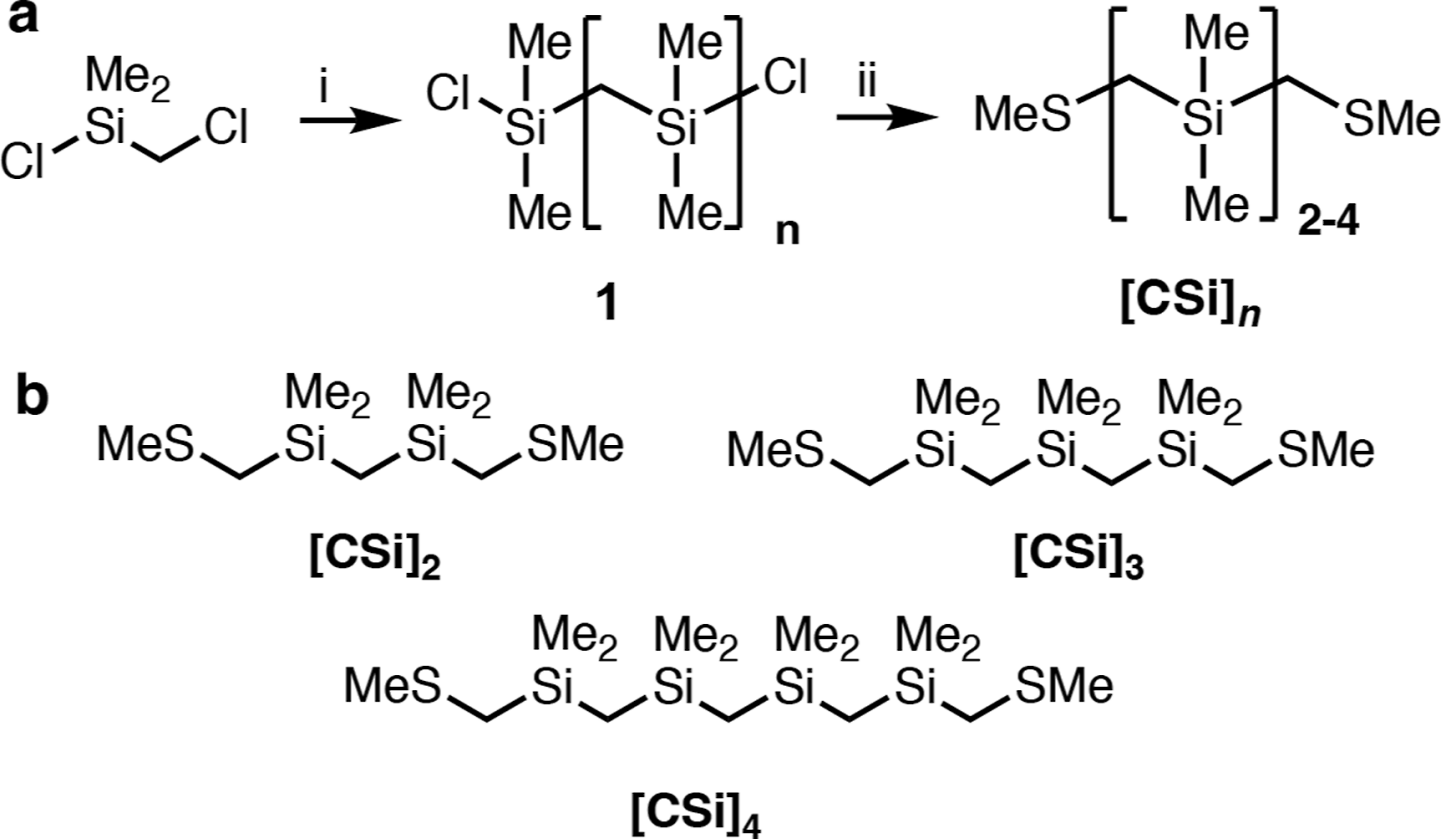
(a) Synthetic scheme for the synthesis of the [**CSi]**_**2–4**_ series. (i) Dichlorodimethylsilane, magnesium, tetrahydrofuran. (ii) CH_3_SCH_2_Li·TMEDA, tetrahydrofuran. Oligomers are separated via recycling preparative gel permeation chromatography. See the Methods section for more detail. (b) Chemical structures of the [**CSi]**_**2–4**_ series.

But what happens if steric influences are removed from the backbone such that all rotational states around a single bond are energetically degenerate? This question motivated us to study carbosilanes **[CSi]**_**2–4**_ (Figure 1b,2) with [CH_2_–SiMe_2_]_*n*_ repeat units in single-molecule junctions. These oligomers are molecular analogues of poly(dimethylsilmethylene) (PDMSM) polymers whose low glass-transition temperatures and characteristic ratios are ascribed to the sterically equivalent methyl and methylene substituents around each Si center (Figure 1b).^31−38^ This article shows that, in the absence of a strong steric bias, intramolecular London dispersion σ-interactions stabilize highly insulating gauche/ortho dihedral conformations in the backbone, even at full junction elongation. This leads us to observe the most insulating molecular junctions that have ever been measured on an experimental junction length basis.^39,40^

## Results and Discussion

**Figure 3:**
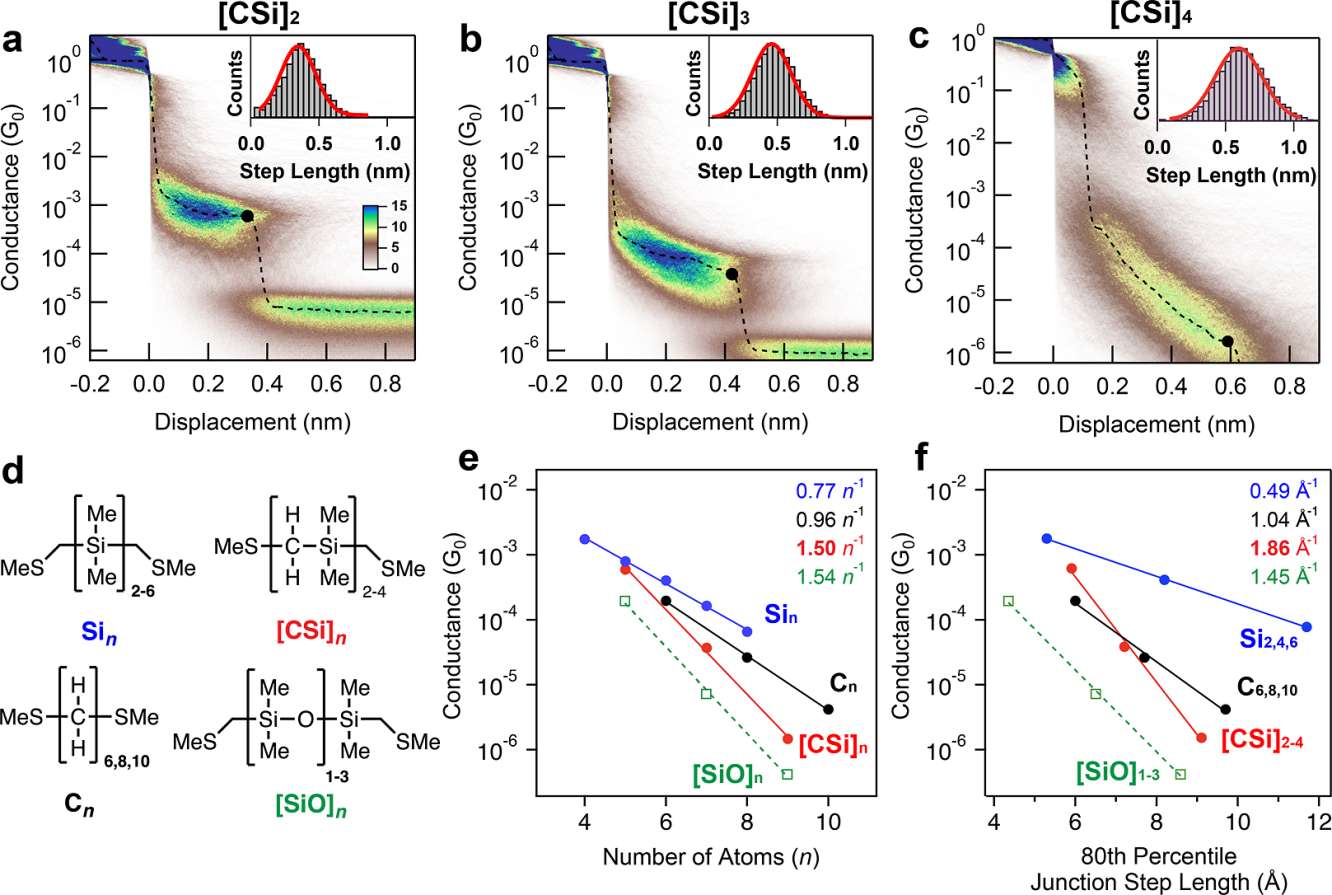
(a–c) 2D conductance-displacement histograms compiling 10,000 measurement traces in 1,2,4-trichlorobenzene solutions of (a) **[CSi]**_**2**_ at 0.1 V bias, (b) **[CSi]**_**3**_ at 0.35 V bias, and (c) **[CSi]**_**4**_ at 1.0 V bias. All histograms were plotted according to the color bar shown in (a), indicating the number of counts per 1000 traces. The dotted black line depicts the most frequently observed conductance at each displacement bin. The black circles indicate the conductance at full junction elongation, where there is a change in the average conductance-displacement slope as described in Figure S3. Inset: step length distribution for all measurement traces. The 50th percentile (most probable) and 80th percentile step lengths are obtained from a Gaussian fit (red curve). (d) Chemical structures for the **Si**_***n***_, **C**_***n***_, **[CSi]**_***n***_, and **[SiO]**_***n***_ wires. (e) Conductance at full junction elongation plotted against the number of backbone atoms between the distal S atoms. β decay constants were obtained by line fitting to the natural logarithm of conductance. β_Si_ = 0.78 ± 0.03 *n*^–1^, β_C_ = 0.96 ± 0.02 *n*^–1^, β_CSi_ = 1.50 ± 0.06 *n*^–1^, and β_SiO_ = 1.54 ± 0.06 *n*^–1^. (f) Conductance at full junction elongation plotted against the 80th percentile junction step length. β_Si_ = 0.49 ± 0.01 Å^–1^, β_C_ = 1.04 ± 0.08 Å^–1^, β_CSi_ = 1.86 ± 0.12 Å^–1^, and β_SiO_ = 1.45 ± 0.05 Å^–1^. The **[SiO]**_**1–3**_ values (green squares) were extracted from ref (40).

We end-functionalized carbosilanes **[CSi]**_**2–4**_ (Figure 2) with gold-binding methylthiomethyl end groups (see Figure S1 and the Methods section for more details)^41−44^ as these linkers form dative contacts with undercoordinated Au atoms that give well-defined conductance profiles.^28,45^ We synthesized alkane **C**_***n***_ (*n* = 6,8,10) and oligosilane **Si**_***n***_ (*n* = 2–6) (Figure 3d) as control compounds according to known procedures.^28,45^ We measured their single-molecule conductance properties using the scanning tunneling microscopy break-junction (STM-BJ) technique (see the Methods section).^46^ Ten thousand STM-BJ measurement traces for each molecule are compiled into a two-dimensional (2D) conductance-displacement histogram (Figure 3a–c, Figure S2).^47,48^ We used a high voltage bias (1.0 V) to measure **[CSi]**_**4**_ to clearly resolve the molecular peak from the noise floor of the instrument. In this specific case, increased voltage bias should not significantly change the conductance value we observe: the HOMO–LUMO gap of this molecule is large, so off-resonant transport should still occur at large applied biases.

We note that **[CSi]**_**3**_ and particularly **[CSi]**_**4**_ have a pronounced conductance-displacement slope, where conductance steadily decreases as displacement increases (Table S1, Figure S2). We thus focus on the average conductance value at full junction elongation (black circles, Figures 3a–c and S2) to compare conductance trends among all series. We define full junction elongation in this context as the point in the 2D histogram where there is a change in conductance-displacement slope near the breakpoint; an example of this analysis is given in Figure S3.^45^ The average conductance values at full elongation for the **[CSi]**_***n***_, **Si**_***n***_, and **C**_***n***_ series are provided in Table 1 and plotted in Figure 3e against *n*, the number of backbone atoms between the distal sulfur atoms in each molecule.

**Table 1:** Molecular Conductance and Junction Displacement Properties

	*n*^a^	G/G_0_^b^	step length (*z*)/nm^c^	Δ*z* per bond/nm^d^	bond length/nm^e^
**C**_**6**_	6	1.96 × 10^–4^	0.36	0.16	0.14
**C**_**8**_	8	2.63 × 10^–5^	0.52		
**C**_**10**_	10	4.18 × 10^–6^	0.67		
**Si**_**2**_^f^	4	1.73 × 10^–3^	0.25	0.24	0.24
**Si**_**4**_	6	4.02 × 10^–4^	0.48		
**Si**_**6**_	8	7.58 × 10^–5^	0.73		
**[CSi]**_**2**_	5	6.08 × 10^–4^	0.35	0.13	0.19
**[CSi]**_**3**_	7	3.78 × 10^–5^	0.46		
**[CSi]**_**4**_	9	1.86 × 10^–6^	0.60		

a Key. Number of atoms between the two S atoms.

b G refers to the most probable conductance at full junction elongation (see Figures S2 and S3).

c Most probable (50th percentile) step length values. The *z* values we obtain for the alkane and silane wires are in good agreement with previous measurements in STM-BJ setups (refs (28 and 50)).

d Average increase in most probable step length across the **C**_***n***_, **Si**_***n***_, and **[CSi]**_***n***_ series as C–C, Si–Si, or C–Si bonds are added to the backbone.

e Average C–C, Si–Si, or C–Si bond lengths in the **C**_***n***_, **Si**_***n***_, and **[CSi]**_***n***_ backbones based on B3LYP-D3/6-311G(d,p) calculations.

f **Si3** and **Si5** are excluded from the table for clarity as the step length trends in the odd oligomers are offset from the even ones due to “odd–even effects”.^29,57−59^

This plot allows us to extract the length-dependent conductance decay parameter (β) value based on the equation, G ∼ e^–β^_n_. Τhe β value describes the strength of coupling between monomers in an oligomeric wire backbone, where low and high β values are ascribed to molecular conductors and insulators, respectively.^49^ The β values we obtain from this approach for the **C**_***n***_ (β_C_ = 0.96 ± 0.02 *n*^–1^) and **Si**_***n***_ (β_Si_ = 0.78 ± 0.03 *n*^–1^) wires are within error of previously reported β values for thiomethyl-terminated alkane (β = 0.94 *n*^–1^) and silane (β = 0.75 *n*^–1^) molecular wires.^29,50−52^

Surprisingly, we find that carbosilane backbones give a far steeper β value (β_CSi_ = 1.50 ± 0.06 *n*^–1^) than purely carbon-based or silicon-based backbones. On a per atom basis, the **[CSi]**_***n***_ β value is within range of the insulating oligo(dimethylsiloxane) **[SiO]**_***n***_ backbones (β_SiO_ = 1.54 *n*^–1^, Figure 2d,e) that give the largest β values reported in the single-molecule literature.^40^ This steep conductance decay becomes more pronounced if we consider how conductance trends with experimentally determined junction length values. In Figure 3f, we plot conductance at full elongation against the 80th percentile step length.^53^ The **[CSi]**_**n**_ series demonstrates a much steeper β value (1.86 Å^–1^) than all other σ-bonded wires, far exceeding that of any other material investigated in the literature including the previously reported **[SiO]**_***n***_ oligo(dimethylsiloxane)s (Figure 3f).^40^ This comparison highlights the potential of oligo(dimethylsilmethylene)s to serve as single-molecule insulators.

The insulating properties of the **[SiO]**_***n***_ series stems from the highly polarized Si^δ+^–O^δ−^ bonds [EN_Si_ = 1.8, EN_O_ = 3.4, electronegativity (EN)] in the backbone that localize molecular orbital density to individual bonds, rather than delocalize across the molecule.^54−56^ While some degree of conductance attenuation is anticipated due to the polarity of the Si^δ+^–C^δ−^ backbones (EN_Si_ = 1.8, EN_C_ = 2.5), the β decay value that we observe experimentally is far greater than what we would expect simply from bond polarity arguments.

This point is captured clearly in our transmission calculations comparing the **C**_***n***_, **Si**_***n***_, and **[CSi]**_***n***_ series. We calculated transmission functions with a density functional theory (DFT)-based nonequilibrium Green’s function method with the PBE exchange-correlation functional^60^ using the AITRANSS^30,31^ postprocessor module within the FHI-aims^61^ package (see the Methods section). While our transmission models of anti **C**_***n***_ (β_C, calc._ = 0.96 *n*^–1^) and transoid **Si**_***n***_ (β_Si, calc._ = 0.76 *n*^–1^) junctions give β values that match our experimental data (Figure S4), we find that all-transoid **[CSi]**_***n***_ junctions give a significantly lower β value (β_SiC, calc._ = 0.99 *n*^–1^) than what we observe experimentally (β_SiC, exp._ = 1.50 *n*^–1^). This discrepancy is the first indicator that, despite the mechanical strain imposed on the carbosilanes upon junction stretching, the all-transoid geometries are not appreciably accessed at full junction elongation.

Indeed, one explanation for the high β value we observe experimentally is that gauche/ortho kinks remain in the backbone upon mechanical elongation (Figure 1b). It has long been appreciated that calculated alkane and silane junctions with gauche-kinked backbones show lower through-bond transmission relative to their anti or transoid conformers.^52,59,62−67^ The introduction of these kinks also significantly lowers transmission in carbosilanes. Indeed, as we will show later in the manuscript, two ortho kinks in the backbone of **[CSi]**_**3**_ lower transmission at the Fermi energy by 200-fold relative to all-transoid conformer.

The experimental step length trends in Table 1 further support the notion that **[CSi]**_***n***_ oligomers maintain kinked backbone geometries at full junction elongation. Junction step length refers to the electrode displacement where a given molecular junction breaks. Though absolute step length values do not map directly with end-to-end molecular lengths due to electrode snapback^68−70^ and linker group effects,^45^ trends in step length provide valuable insights into the structural details of molecular junctions. The step lengths for all traces are plotted in the insets of Figure 3 and Figure S2, where a Gaussian fit gives the most probable step length value (*z*, Table 1). In Table 1, Δ*z* denotes the average increase in *z* as C–C, Si–Si, or C–Si bonds are successively added to **C**_**6**_, **Si**_**2**_, and **[CSi]**_**2**_. We find that the Δ*z* and bond length values are quite similar for the anti alkanes as well as transoid silanes, while the Δ*z* value is much shorter than the C–Si bond length for the carbosilanes. Crucially, we find that carbosilane junctions (Δ*z* = 0.13 nm) exhibit a much smaller Δ*z* than alkane junctions (Δ*z* = 0.16 nm), though C–Si bond lengths (0.19 nm) are much longer than the C–C bonds (0.14 nm) in alkanes. Previous studies on PDMSMs indicate that the CSiC and SiCSi bond angles are rigid and tetrahedral;^34,35,71^ the only significant source of conformational flexibility is backbone torsion. As these kinked structures give shorter step lengths than fully transoid structures, the step length trends lead us to infer that gauche or ortho dihedral geometries persist in the backbone as the junction is stretched.

Three questions emerge from our central hypothesis that carbosilane junctions maintain coiled geometries upon mechanical stretching: (1) What is the energetic driving force that would cause kinked [CH_2_–SiMe_2_]_*n*_ backbones to be favored over transoid ones? (2) Why is this effect absent in the –CH_2_– (**C**_***n***_) and –SiMe_2_– (**Si**_***n***_) backbones? (3) If the σ-bonded backbone is colinear with the elongation axis, why does the mechanical strain of junction stretching not straighten the backbone to its longest transoid geometry? The DFT calculations below provide rationalization for these effects based on intramolecular London dispersion interactions.

**Figure 4:**
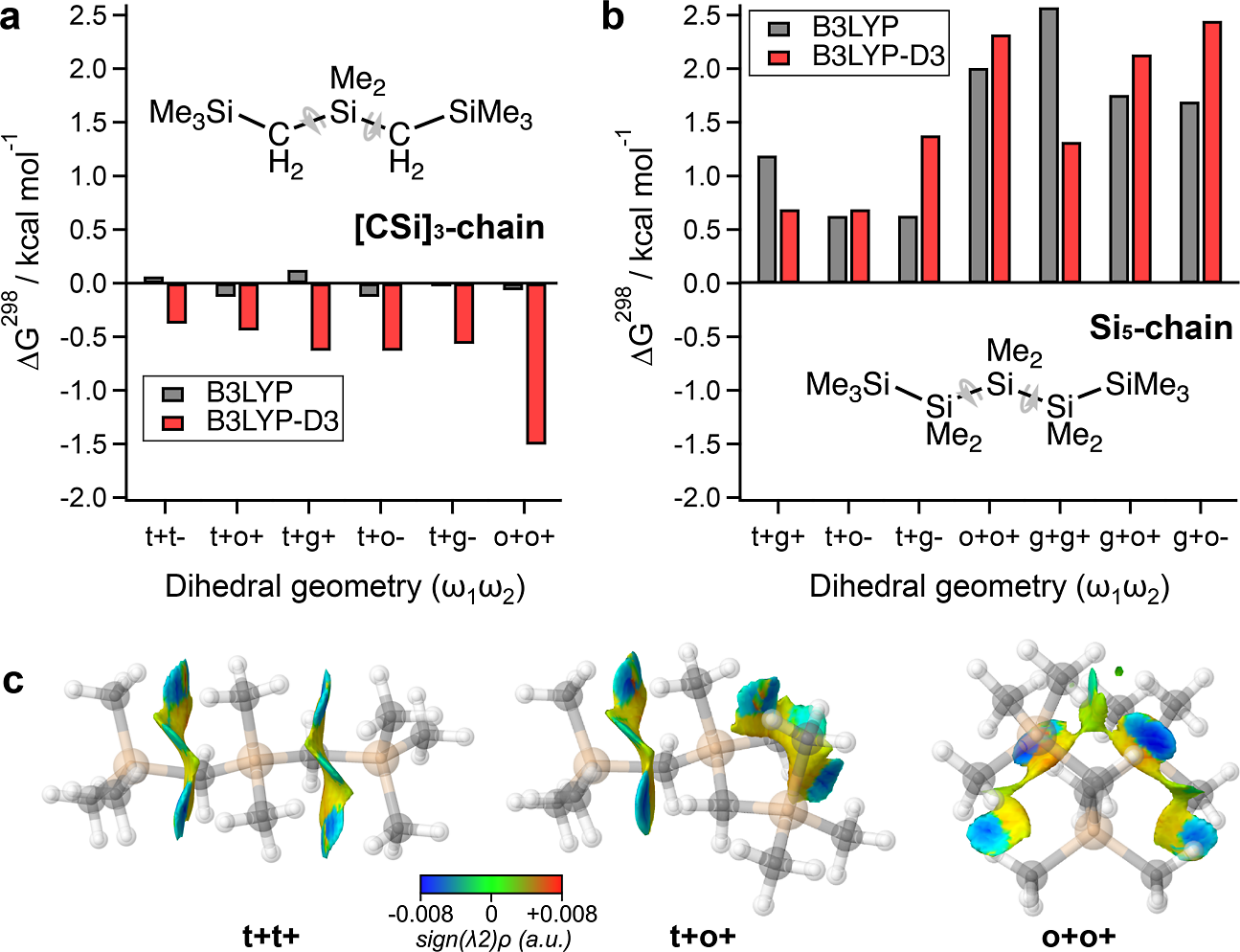
(a,b) Free energies (ΔG^298^) of **[CSi]**_**3**_**-chain** (a) and **Si**_**5**_**-chain** (b) conformers plotted relative to their respective t^+^t^+^ conformers. Structures were optimized at B3LYP/6-311G(d,p) (gray bars) or B3LYP-D3/6-311G(d,p) (red bars) without constraint. We classify dihedral structures as follows: gauche (ω = ± 47–55°), ortho (ω = ± 67–114°), or transoid (ω = ± 141–172°). Only conformers that correspond to minima on the potential energy surface are included. The dihedral angles and energy values for the **[CSi]**_**3**_**-chain** conformers are provided in Table 1. (**c**) NCI plots for the t^+^t^+^_,_ t^+^o^+^, and o^+^o^+^ conformers of **[CSi]**_**3**_**-chain** optimized at the B3LYP-D3/6-311G(d,p) level. NCI surface areas plotted at *s* = 0.7 au cutoff and −0.02 < ρ < 0.02 au for SCF densities. We apply a narrower color scale to emphasize strong intramolecular dispersion interactions in blue.

Despite extensive experimental and theoretical studies of PDMSM backbones in the polymer literature, a preference for kinked geometries has never been described. While most PDMSM conformational studies have been executed via molecular dynamics simulation,^33,34,38^ Raptis and Melissas used DFT calculations (B3LYP/6-311G) to show that short PDMSM oligomer geometries are defined by transoid (t^±^) and ortho/gauche (o^±^, g^±^) minima that are energetically degenerate.^35^ Crucially, the B3LYP hybrid functional used in this study did not account for intramolecular dispersion interactions. DFT corrections such as Grimme’s D3 approach are necessary to account for dispersion in hybrid functionals.^72,73^ The impact of dispersion interactions is often quantified by evaluating relative energy differences between B3LYP and B3LYP-D3 calculations of the same set of structures.^1,2,74^ In Figure 4a,b, we apply this strategy to compare the energies of conformational minima in the linkerless **[CSi]**_**3**_**-chain** and **Si**_**5**_**-chain** analogues against their all-transoid (t^+^t^+^) rotamers. As the only molecular differences distinguishing **[CSi]**_**2**_ from **[CSi]**_**4**_ are the additions of backbone C–Si bonds, we focus on the linkerless σ-chains to minimize complexity and simplify our analysis. Their specific dihedral geometries, end-to-end distances, and energies are also provided in Tables S2 and S3 (Supporting Information).

In line with Raptis and Melissas’ finding, our DFT studies of **[CSi]**_**3**_**-chain** show that the kinked geometries are isoenergetic with the t^+^t^+^ configuration when we similarly omit the D3 correction (gray bars, Figure 4a). However, calculations with the B3LYP-D3 functional indicate that the t^+^t^+^ rotamer is the highest energy conformer (red bars, Figure 4a); all other geometries are lower in free energy, with the o^+^o^+^ rotamer being the most stable (−1.5 kcal mol^–1^ relative to t^+^t^+^). This difference indicates that dispersion interactions strongly favor coiled geometries in oligo(dimethylsilmethylene) chains. In contrast, applying the same treatment to **Si**_**5**_**-chain** gives the t^+^t^+^ configuration as the lowest energy conformer regardless of whether dispersion interactions are accounted for (Figure 4b), since the strong steric influence of the SiMe_2_Si group biases oligosilane backbone geometries toward transoid conformations (Figure 1a).^75^ The alkane series resembles what is seen in the silanes.

We performed NCIPLOT calculations^76^ on the B3LYP-D3-optimized t^+^t^+^, t^+^o^+^, and o^+^o^+^ conformers of **[CSi]**_**3**_**-chain** to visualize where these stabilizing London dispersion interactions occur in the backbone (Figure 4c). While there are CH···H interactions between vicinal Si(CH_3_)_2_ groups in the t^+^t^+^ conformer, these are comparatively weaker than the coiled dispersion interactions occurring in the o^+^o^+^ conformer [*s*(ρ) plots are provided in Figure S5]. The two strongest CH···H interactions (dark blue, Figure 4c) in o^+^o^+^ occur between backbone CH_2_ and peripheral Si(CH_3_)_2_ moieties. Additional H···H interactions occur between the first and second (light blue) and first and third (green) SiMe_2_ moieties. These interactions are explicitly depicted in Figure 1b Newman projections.

The o^+^o^+^ dispersion interactions are cooperative in the sense that they occur only when both dihedrals are ortho-disposed. In other words, breaking one strong contact by rotating to a transoid dihedral means that the other strong contact must also be broken. This creates an intramolecular stapling effect that enforces kinked geometries, leading to a 3-fold energetic stabilization for the o^+^o^+^ geometry compared to the t^+^o^+^ one. This difference in interdependency can be visualized in Figure 4c: the NCI interactions in the t^+^t^+^ and t^+^o^+^ conformers are separated into two independent strips of isosurface density but are joined together into one interconnected strip of NCI density for the o^+^o^+^ conformer.

**Figure 5:**
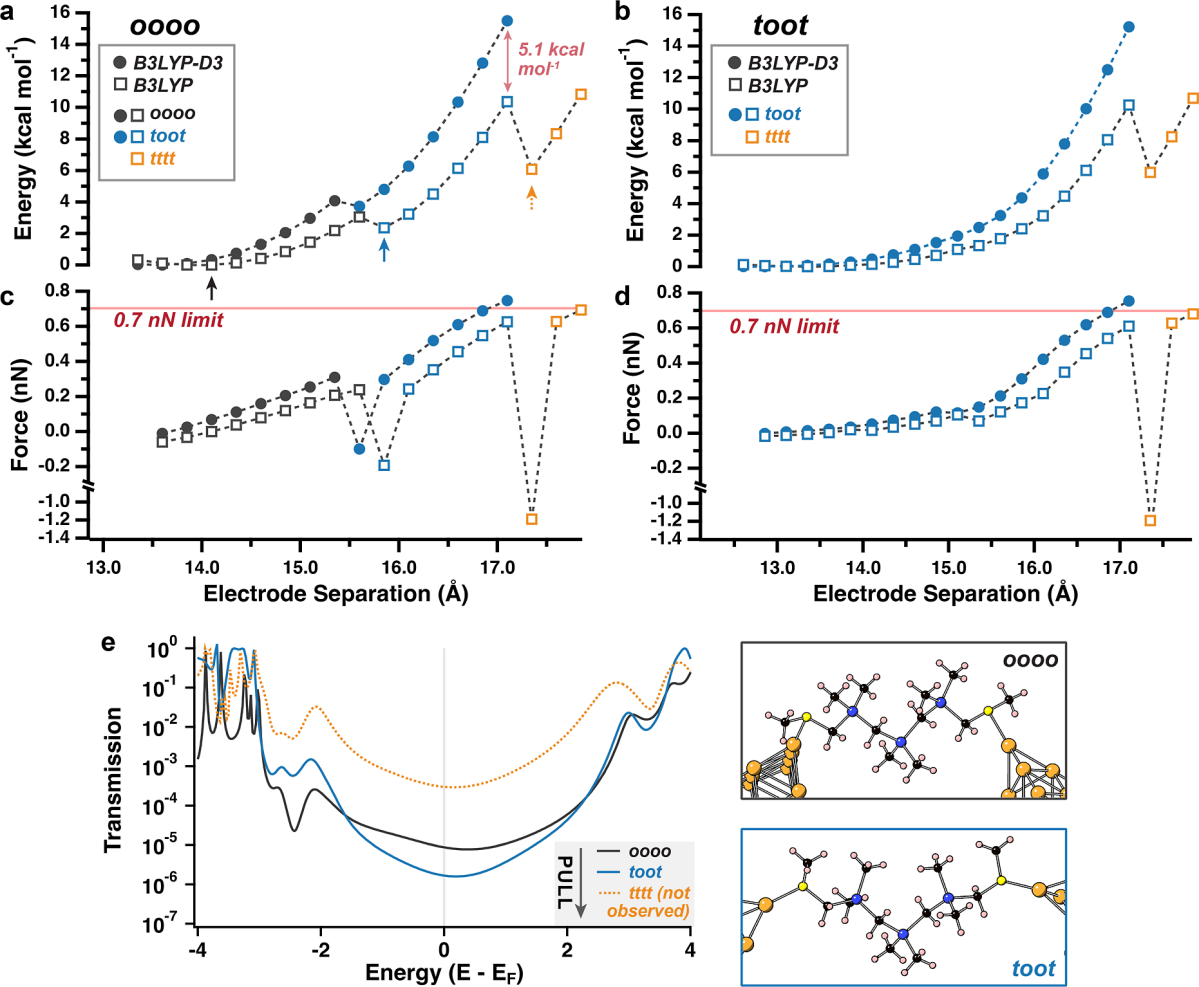
(a,b) Junction elongation plots track change in energy and backbone conformation as (a) oooo and (b) toot **[CSi]**_**3**_ initial geometries are stretched between Au_10_ pyramids until the junction breaks. Energies are plotted relative to the initial geometry optimized with either the B3LYP-D3 (filled circle) or the B3LYP (empty square) functional. Color changes indicate a major change in the backbone geometry. (c,d) Junction pulling force calculated from the change in energy with respect to electrode separation. Acute drops in force correspond to major configuration changes in the backbone dihedral geometries. (e) Transmission calculations and structures of Au_20_-molecule-Au_20_ junctions with molecular geometries extracted from select points [marked by arrows in (a)] along the oooo pulling trajectories. The light gray vertical line is plotted at E_F_ as a visual aid. General note: all ortho (o) and transoid (t) conformers refer to o^+^ or t^+^ dihedral geometries.

While it is a combination of entropic^77^ and dispersion effects that lead to coiled carbosilane geometries being picked up in the junction initially (see Supporting Note 1), our findings suggest that intramolecular dispersion forces can keep coiled carbosilane geometries intact in spite of the mechanical strain applied along the backbone as the junction is stretched. To investigate this notion computationally, we modeled the stretching of coiled junctions between Au_10_ electrodes to see whether the internal backbone kinks unravel to all-transoid states before a breaking force limit is reached (Figures 5, S6–S8). Conductive atomic force microscopy measurements have shown that thiomethyl-terminated alkane wires sustain average pulling forces up to 0.7 nN before the dative R_2_S–Au linkages break and junction rupture occurs.^78,79^ Our DFT calculations indicate that the donor–acceptor R_2_S–Au bond strengths are virtually identical between the previously reported^78^ thiomethyl-terminated butane (14.7 kcal mol^–1^) and the **[CSi]**_**3**_ geometries discussed herein (13.8–14.7 kcal mol^–1^, Table S4). These similarities suggest that the Au–S dative bonds in the **[CSi]**_**n**_ series can withstand average junction stretching forces up to 0.7 nN.

We note that there are many more low-lying conformers for **[CSi]**_**3**_ junctions than the **[CSi]**_**3**_**-chain** models as shown in Figure 4 as there are now eight internal dihedrals, each with their own set of conformational states (see Supporting Note 2). Many of these conformers likely exist in the junction’s overall conformational landscape and contribute to the observed conductance behavior and trends. While a comprehensive investigation of all possible junction geometries and their trajectories is outside the scope of this work, we carry out a focused analysis on representative geometries that help illustrate how dispersion can impact the evolution of molecular geometry and electronic transmission as carbosilanes are stretched to a 0.7 nN force limit (Figure 5).

We focus on the initial geometries of **[CSi]**_**3**_ with central o^+^o^+^ dihedral configurations as Figure 4 indicates that these internal geometries are the most stabilized by dispersion. Figures 5 and S6 plot the pulling trajectories of initial o^+^o^+^o^+^o^+^, t^+^o^+^o^+^t^+^, or o^+^o^+^o^+^t^+^ junction geometries (see Supporting Note 2 for more geometry details). For each conformer, we performed DFT calculations at the B3LYP/6-31G(d,p)/def2-SVP(Au) level^47^ with and without Grimme’s D3 correction (filled circles and empty squares in Figure 5a–d, respectively) to model how junction geometry, energy, and applied force are impacted by dispersion interactions upon junction stretching. Meanwhile, Figure 5e models transmission at the zero-energy point or after a major change in internal dihedral geometry occurs (arrows in Figure 5a). We compare transmission calculations on structures with and without dispersion corrections invoked during geometry optimization as shown in Figure S9. In most modeled geometries, we find only marginal differences in transmission at the Fermi energy (E_F_). We note that in some cases intramolecular dispersion interactions can alter molecular geometry in ways that lead to the onset of destructive quantum interference antiresonance features near E_F_ (Figure S9d). The full details of these calculations are provided in the Methods section.

Broadly, we find that the dispersion correction increases both the relative energy and the force required to stretch molecular junctions as the Au–Au distance widens, likely due to the energetic penalty from disrupting the London dispersion interactions that require short contact.

Junction stretching of the o^+^o^+^o^+^o^+^ conformer leads to a conformational transition to t^+^o^+^o^+^t^+^ that is accompanied by an acute drop in energy and force (Figure 5a,c). This transition occurs regardless of whether dispersion corrections are included. Figure 5e shows that these outer ortho to transoid dihedral transitions give a modest decrease in transmission at the Fermi energy (E_F_), which is consistent with the small dip in most frequent conductance that we observe experimentally starting around ∼0.3 nm as shown in Figure 3b. In the absence of Grimme’s dispersion correction, we find that further junction stretching leads to a geometry change from the t^+^o^+^o^+^t^+^ (blue square) to t^+^t^+^t^+^t^+^ (orange square) configuration before the 0.7 nN limit is reached. If this change to the all-transoid geometry did occur, we would expect to find an increase in transmission by 2 orders of magnitude in the last ∼1 Å prior to its breakpoint (dotted orange line, Figure 5e). This is not consistent with what we observe experimentally in Figure 3a–c. When dispersion interactions are accounted for, we instead find that the t^+^o^+^o^+^t^+^ junction geometry is maintained past the 0.7 nN force threshold as the fully elongated, dispersion-corrected geometry at the calculated breakpoint is 5.1 kcal mol^–1^ higher in relative energy than the equivalent point without the dispersion correction (Figure 5a). This suggests that the Au-linker contact breaks before the internal ortho staples are disrupted. The same general trends are observed with an initial t^+^o^+^o^+^t^+^ junction geometry (Figure 5b,d), where the dispersion-corrected junction stretching trajectory ends with the poorly transmissive t^+^o^+^o^+^t^+^ dihedral configuration rather than the all-transoid configuration. These findings suggest that London dispersion interactions such as the ones shown in Figure 4c may enable kinked geometries to be sustained even at full junction elongation, where mechanical force is highest.

We observe a different yet experimentally consistent outcome when we invoke an initial o^+^o^+^o^+^t^+^ geometry (Figure S6). Regardless of dispersion, a transition to the o^+^t^+^o^+^t^+^ junction configuration occurs. This geometry is sustained upon elongation without a switch to the all-transoid configuration. This transition is also accompanied by a mild decrease in transmission (Figure S6c) that is consistent with the decreasing conductance we observe upon junction elongation. Thus, in all three models studied here, we find that internal ortho dihedrals persist in the backbone at junction breakdown.

Finally, we modeled elongation in Au_10_–**Si**_**5**_–Au_10_ junctions with a starting o^+^o^+^o^+^o^+^ configuration as a control (Figure S7). Regardless of whether dispersion interactions are accounted for, these junctions reach fully transoid geometries upon junction stretching prior to the 0.7 nN force limit as the dispersion interactions in permethylated oligosilanes are outweighed by steric influences. This concept reinforces that in the absence of strong steric biases intramolecular dispersion can play a dominant role in dictating molecular geometry in σ-bonded molecular junctions.

## Conclusion

Intramolecular London dispersion σ-interactions have been characterized extensively in ensemble spectroscopic measurements.^80,81^ To the best of our knowledge, the present work is the first instance where these specific intramolecular interactions have been observed in a single-molecule context. These studies, along with related work by Venkataraman, Hybertsen, and co-workers investigating molecule-electrode van der Waals interactions,^82^ point to mechanical break-junction systems as useful tools to deepen our understanding of London dispersion forces where trends in conductance, step length, and mechanical force measurements provide physical readouts for the presence and strength of dispersion interactions in single-molecule circuits.

Finally, this work highlights the untapped potential of using intramolecular London dispersion interactions to engineer single-molecule electronics. Here, we show one specific use case, where London dispersion interactions structurally enforce kinked junction geometries that annul quantum transport and give single-molecule insulators with the highest experimental β values reported to date. However, it should be possible to apply London dispersion design concepts to deliberately install other features or functions into molecular junctions. For example, one can imagine using London dispersion interactions to instead enforce highly conductive yet sterically disfavored junction conformations, reversibly disrupt and restore these dispersion interactions with an external stimulus for switchable molecular electronic components,^11−14^ or perform novel molecular electronic functions.

## Methods

### Synthesis

General synthesis and characterization information and NMR spectra are provided in the Supporting Information. The synthesis for these compounds were adapted from previously reported procedures^29,83^ and executed according to Figure 2. An oven-dried 250 mL three-necked flask equipped with a 100 mL addition funnel, reflux condenser, septa, and stir bar was connected to a Schlenk manifold under a nitrogen atmosphere. The flask was charged with magnesium turnings (0.69 g, 28.42 mmol, 1.00 equiv). The addition funnel was filled with a mixture of dichlorodimethylsilane (5.50 g, 42.62 mmol, 1.50 equiv) and chloro-(chloromethyl)dimethylsilane (4.07 g, 28.42 mmol, 1.00 equiv) in 80 mL of tetrahydrofuran (THF). This mixture was added dropwise at room temperature over the course of 5 h followed by overnight reflux. The next day, THF was removed in vacuo and 100 mL of anhydrous pentane was added to the flask. Salts were filtered over dry Celite using an air-free Schlenk filter into a 250 mL Schlenk flask. The solution was concentrated in vacuo to give 2.02 g of clear, colorless oil **1** (Figure 2) that was carried forward without further purification.

A separate oven-dried 50 mL Schlenk flask was equipped with a stir bar and rubber septa, then connected to a Schlenk manifold and charged with 2.5 M *n*-butyllithium solution in hexanes (4.60 mL, 62.52 mmol, 2.20 equiv). The Schlenk flask was cooled to 0 °C. N,N,N′,N′-Tetramethylethylenediamine (7.27 g, 62.52 mmol, 2.20 equiv) was added dropwise via a syringe, and the mixture was diluted with 3 mL of anhydrous pentane and stirred for 20 min. Dimethyl sulfide (3.89 g, 62.52 mmol, 2.20 equiv) was added dropwise over 5 min, then stirred for 4 h at room temperature. The flask containing **1** was dissolved in THF (110 mL) and cooled to 0 °C. The dimethyl sulfide solution was cannulated into the flask and stirred overnight. The next day, the reaction was quenched with methanol (10 mL) and the solvent was removed through rotary evaporation. The resulting yellow oil was redissolved in hexane and filtered through a silica plug using a 1:1 mixture of dichoromethane/hexane to yield 1.41 g of colorless oil. This was further purified via preparative gel permeation chromatography using a JAI Labo-Ace LC-5060 equipped with a JAIGEL-2.5 HR and JAIGEL-2 HR gel permeation chromatography column in series using *n*-hexane (95%, Avantor) as the eluent. A characteristic chromatograph is shown in Figure S1. The following compounds were isolated:

**[CSi]**_**2**_ colorless oil (324 mg, 4.5% yield) ^1^H NMR (600 MHz, CDCl_3_): δ 2.15 (s, 6H), 1.80 (s, 4H), 0.13 (s, 12H), −0.06 (s, 2H). ^13^C NMR (151 MHz, CDCl_3_): δ 23.32, 21.00, 1.44. ^29^Si NMR (79 MHz, CDCl_3_): δ 1.16. HRMS (TOF MS ASAP+) for [C_9_H_24_S_2_Si_2_]: calculated = 237.0623 found = 237.0623 [M – CH_3_]^+^

**[CSi]**_**3**_ colorless oil (159 mg, 1.72% yield) ^1^H NMR (600 MHz, CDCl_3_): δ 2.15 (s, 6H), 1.78 (s, 4H), 0.13 (s, 12H), 0.08 (s, 6H), −0.12 (s, 4H). ^13^C NMR (151 MHz, CDCl_3_): δ: 23.41, 20.88, 4.39, 2.85. ^29^Si NMR (79 MHz, CDCl_3_): δ 0.91, 0.79. HRMS (TOF MS ASAP+): for [C_12_H_32_S_2_Si_3_], calculated 309.1018; found, 309.1023 [M – CH_3_]^+^

**[CSi]**_**4**_ colorless oil (47 mg, 0.42% yield) ^1^H NMR (600 MHz, CDCl_3_): δ 2.15, 1.78, 0.12, 0.07, −0.15, −0.20. ^13^C NMR (151 MHz, CDCl_3_): δ: 23.44, 20.87, 7.56, 4.57, 2.96. ^29^Si NMR (119 MHz, CDCl_3_): δ 0.53, 0.15. HRMS (TOF MS ASAP+): for [C_15_H_40_S_2_Si_4_], calculated 381.1414; found, 381.1418 [M – CH_3_]^+^

### STM-BJ Measurements

Details of the STM-BJ technique have been reported elsewhere.^46^ In brief, a gold STM tip is brought in and out of contact with a gold substrate over a dilute solution of the target molecule (1 mM in 1,2,4-trichlorobenzene solvent) while recording the electrical current and displacement between the electrodes. The electrodes are first brought into contact by pushing the tip into the substrate until a conductance of 5 *G*_0_ is achieved, then retracted at a rate of 20 nm/s for 5 nm. A single Au–Au point contact has a conductance (*G*) of 1 *G*_0_ (=2*e*^2^/*h*), which serves as our fundamental unit of conductance. Once the point contact breaks, a proximal target molecule may bridge the electrodes to form a single-molecule junction. The junction is stretched by separating the electrodes until the Au-molecule-Au junction ruptures, thus ending a single measurement trace. 2D conductance-distance histograms are generated using 100 bins/decade for conductance (*y* axis) and 1000 bins/nm along the displacement axis (*x* axis) and aligned at 0 nm displacement and 0.5 *G*_0_.

STM break-junction measurements were acquired on a home-built instrument with custom software and electronics. A PI 840.10 linear piezo (Physik Instrumente) controlled by a 24 bit NI-4461 DAQ module (National Instruments) is used for precise control of the tip electrode. Conductance measurements are conducted at a rate of 40 kHz using a FEMTO DLPCA-200 amplifier (FEMTO Messtechnik GmbH). A resistor in series with the amplifier and the molecular junction is used to set the sensitivity of the amplifier. Series resistor and voltage bias values are given in Figure S2 caption. Gold-on-steel substrates were prepared by polishing stainless 10 mm AFM/STM specimen discs (Ted Pella) using a hand rotary tool followed by ultrasonic cleaning. The discs were arranged in an Angstrom NextDep Thermal Evaporator and 200 nm of gold (Thermo Scientific Chemicals, 99.95% purity) were deposited onto the surface of the discs. Gold STM tips were formed by cutting gold wire (Alfa Aesar, 0.25 mm, 99.998% purity). Prior to measurement, both the gold tips and wires were cleaned via ozonolysis using an UV ozone generator (Novascan) for 30 min. Solutions of target molecules were made in 1,2,4-trichlorobenzene (>98%, TCI America). Measurements were done in both 1 mM and 0.1 mM 1,2,4-trichlorobenzene solutions, but we did not observe any significant variance in outcome.

### Calculations

#### Thermochemical Calculations

Energies, Δ*G*^298^ values, molecular dihedral angles, and molecular lengths of the molecules described in Figure 4 and Tables S1–S4 were obtained from gas-phase DFT calculations via Gaussian16.^84^ Molecular geometry optimizations were carried out at the B3LYP/6-311G(d,p) or B3LYP-D3/6-311G(d,p) level unless otherwise noted in the caption. In all frequency calculations, every stationary point was identified as a minimum with no imaginary frequencies observed. NCIPLOT calculations were carried out from B3LYP-D3/6-311G(d,p)-optimized structures. Figure S5 plot was obtained from NCIPLOT;^76^ the visual representations in Figure 4 were created in Jmol.

#### Junction Pulling Simulations

We first optimized the geometry of **[CSi]**_**3**_ and **Si**_**5**_ free molecules without constraint from initial dihedral angles of 165° (t^+^) or 80° (o^+^) for the listed internal backbone torsions and anti geometries for all other backbone dihedrals (Supporting Note 2). Rigid Au_10_ pyramids were appended to each S atom in the optimized free molecule. We use fixed Au_10_ pyramids as proxies for the electrodes at the B3LYP/6-31G(d,p)/def2-SVP(Au) level,^47^ with and without Grimme’s D3 correction.^72^ We note that using simpler Au_2_ diatoms as electrodes reproduce the same qualitative effect (Figure S8).

The Au_10_-molecule-Au_10_ system was relaxed without any other constraints to obtain our starting geometries for the junction stretching simulations. From these geometries, we either shortened or extended the distance between the Au apex atoms in 0.25 Å increments, allowing the molecule to relax at each stage between the fixed Au–Au distances to simulate junction compression or stretching. The energies of these constrained junction geometries are plotted relative to the energy of the optimized Au_10_-molecule-Au_10_ without the distance constraint. In these calculations, we define the junction breakpoint as the point where the system crosses the 0.7 nN force limit or the molecule detaches from the Au_10_ pyramid. We obtained stretching force by taking the derivative of energy with respect to Au–Au displacement.^45^

Select geometries (with and without D3 correction) from the pulling trajectory were extracted for subsequent transmission calculation. We replaced the Au_10_ clusters with Au_20_ clusters without changing any other details of the B3LYP-optimized molecular geometry. We calculated the transmission function with a DFT-based nonequilibrium Green’s function method with the PBE exchange-correlation functional^60^ and a light (double-ζ equivalent) basis set using the AITRANSS^30,31^ postprocessor module within the FHI-aims^61^ package to obtain transmission function plots.

#### Transmission β Value Plots

Single Au atoms are appended to the linker groups of molecular models with the following initial geometries: S–Au length of 2.49 Å, C–S–Au angle of 110°, Si–C–S–Au dihedral of 180°, and Si–C–S–Me dihedral of 90°. These models undergo an initial optimization with a maximum residual force per atom of 0.01 eV Å^–1^. Au_20_ pyramids are added to each Au atom to approximate the Au(111) structures. These junctions were used to calculate the molecule’s energy-dependent transmission function according to the approach described above to give the plots as shown in Figure S4.

## Supporting Information Available

Additional figures, tables, notes, structural characterization, STM-BJ measurement details, and DFT details (PDF)
