# Supplementary material for: Intramolecular London Dispersion Interactions in Single-Molecule Junctions
Source: J Am Chem Soc. 2024 Feb 7;146(7):4716–26. doi: 10.1021/jacs.3c12183 (PMC10885141; doi:10.1021/jacs.3c12183)
Supplement: Supplementary file 1 — ja3c12183_si_001.pdf [file ja3c12183_si_001.pdf]

Supporting Information for

Intramolecular London Dispersion Interactions in Single-Molecule Junctions

Matthew O. Hight,[1] *Joshua Y. Wong,*[1] *Ashley E. Pimentel,*[1] *Timothy A. Su*[1,2]*

[1]Department of Chemistry, University of California, Riverside, California 92521
[2]Materials Science and Engineering Program, University of California, Riverside, California 92521

Table of Contents

I. Supporting Figures, Tables, and Notes S-2

II. Synthetic Procedures and Characterization of Compounds S-13
1. General Synthesis and Characterization Information S-13
2. NMR Spectra S-14

III. Supporting Information References S-19

I. Supporting Figures, Tables, Notes

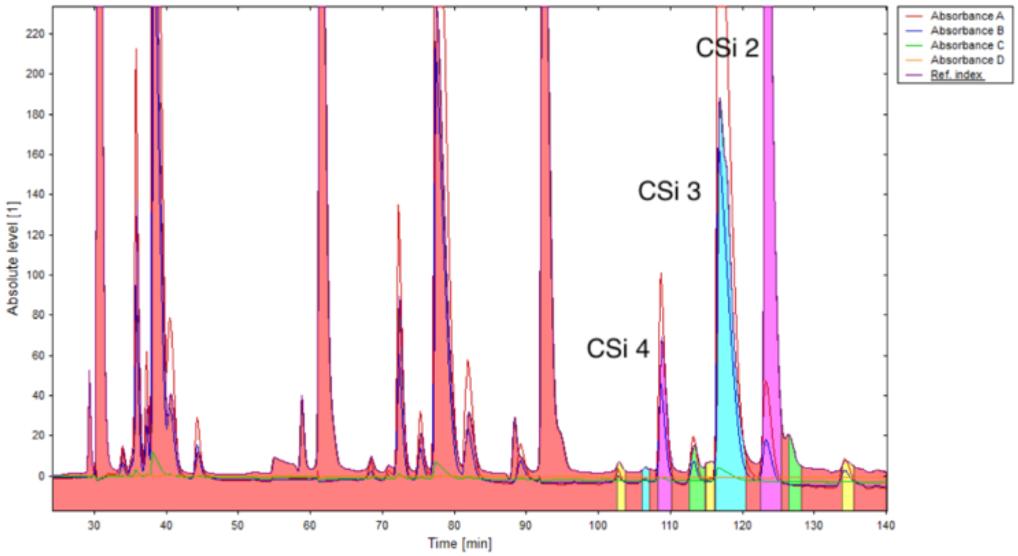

Supporting Figure S1. A representative recycling preparative gel permeation chromatogram to separate the **[CSi]$_{2\text{-}4}$** oligomers in *n*-hexane with JAIGEL 2 HR and 2.5 HR columns in series. Absorbance A = 206 nm, Absorbance B = 220 nm, Absorbance C = 254 nm, Absorbance D = 280 nm.

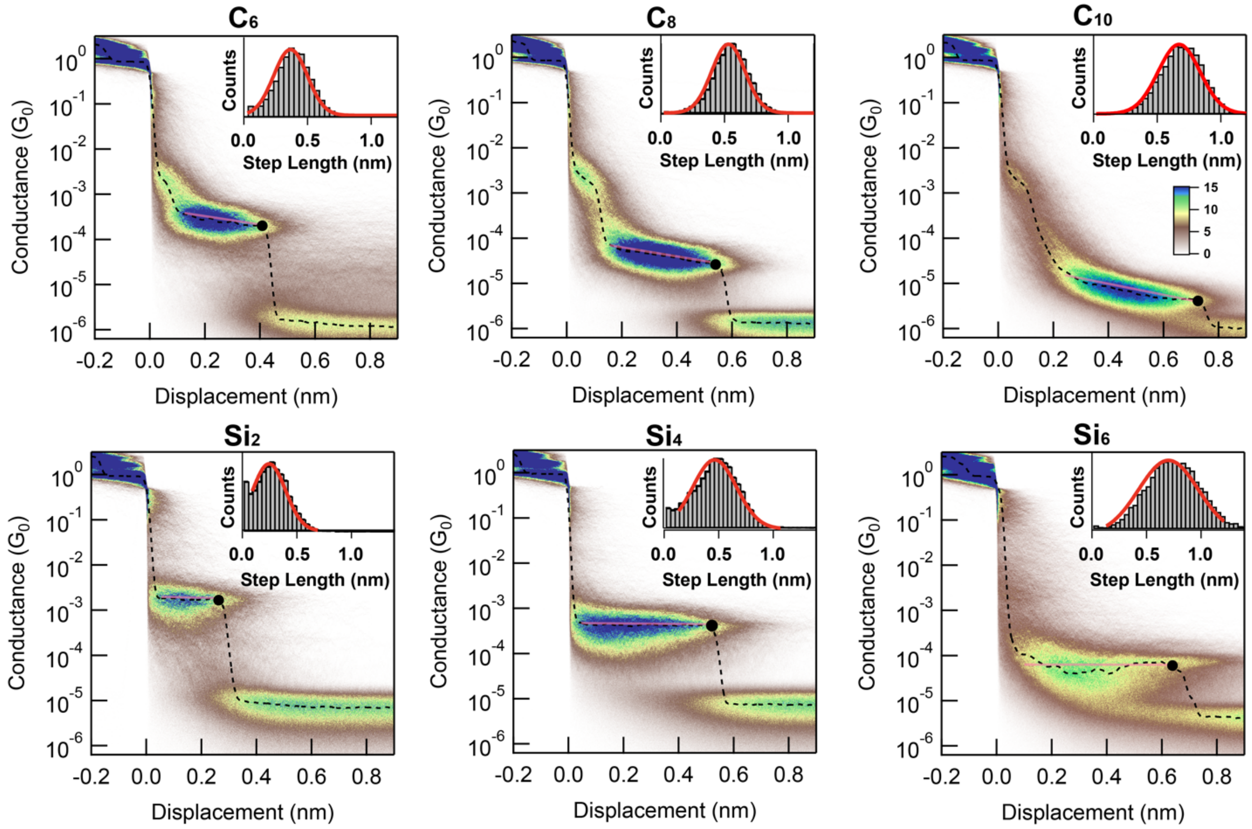

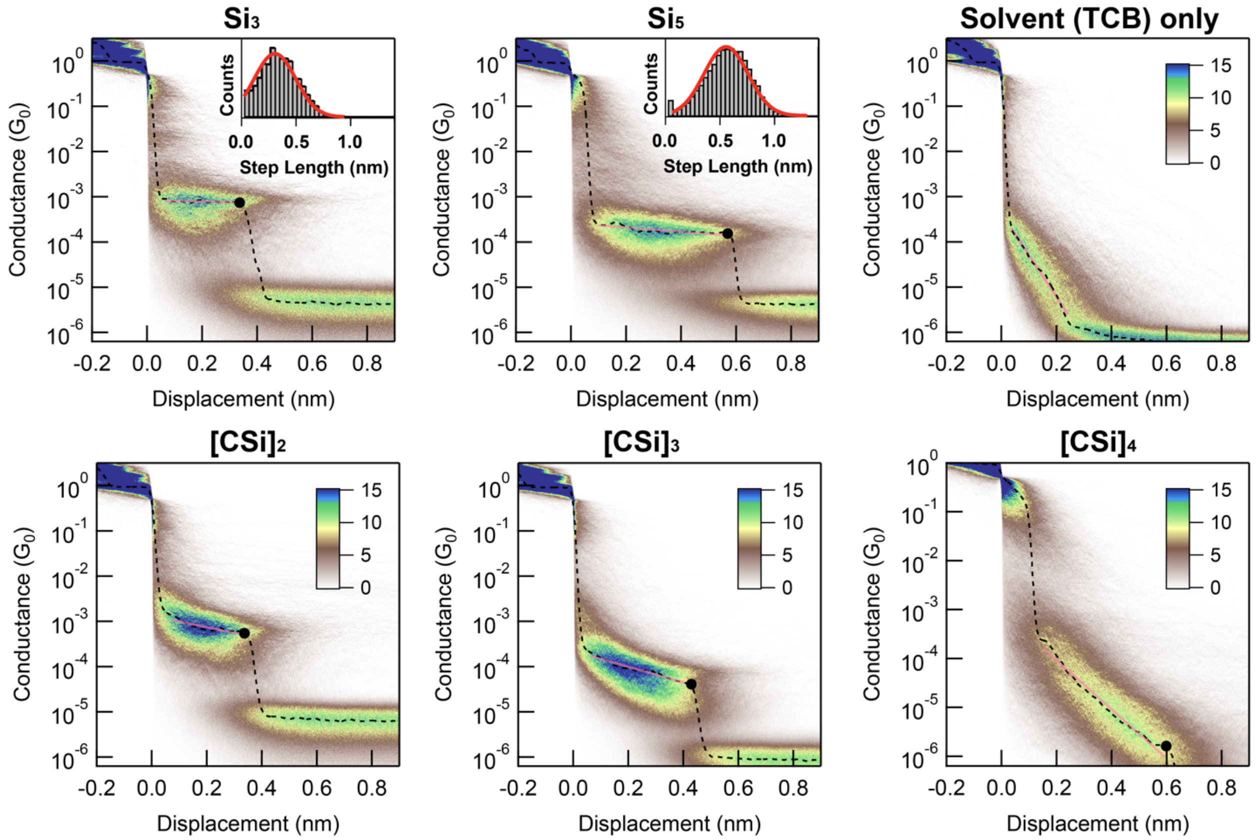

Supporting Figure S2. Two-dimensional conductance-displacement histogram compiling 5,000 to 10,000 measurement traces of **C_6** (1 mM, 0.35 V bias, 1 MΩ resistor), **C_8** (1 mM, 0.35 V bias, 1 MΩ resistor), **C_{10}** (1 mM, 0.35 V bias, 1 MΩ resistor), **Si_2** (1 mM, 0.1 V bias, 97 kΩ resistor), **Si_4** (1 mM, 0.1 V bias, 97 kΩ resistor), **Si_6** (1 mM, 0.1 V bias, 97 kΩ resistor), **Si_3** (1 mM, 0.1 V bias, 97 kΩ resistor), **Si_5** (1 mM, 0.1 V bias, 97 kΩ resistor), **TCB only** (0.1 V bias, 97 kΩ resistor), **$[CSi]_2$** (1 mM, 0.1 V bias, 97 kΩ resistor), **$[CSi]_3$** (1 mM, 0.35 V bias, 1 MΩ resistor), **$[CSi]_4$** (0.1 mM, 1.0 V bias, 1 MΩ resistor) in 1,2,4-trichlorobenzene solutions. All histograms plotted to a maximum of 15 counts per 1000 traces. The dotted black line depicts the most frequently observed conductance at each displacement bin. The black circles indicate the conductance at full junction elongation, which we define as the point at which the dotted black line changes in slope. The pink line marks the line fit of the conductance-displacement slope between the junction startpoint and breakpoint. We define these two points from the derivative of the average conductance-displacement curve (dotted black line, see **Figure S3** for more detail). Inset: step length distribution for all measurement traces. The 50th percentile (most probable) and 80th percentile step length is obtained from a Gaussian fit (red curve).

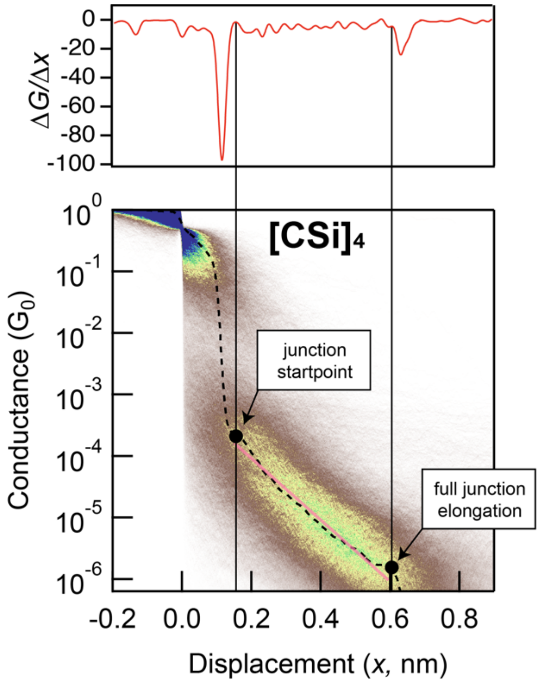

Supporting Figure S3. The derivative of average conductance (*G*) as a function of displacement (*x*) is plotted (top, red curve) to identify the average point where the slope changes in the conductance-displacement histogram (bottom, dotted line) that signifies the average junction startpoint and breakpoint at full junction elongation. The average conductance at this displacement value (right black circle) gives the most probable conductance values at full junction elongation in Table 1. The β_{slope} value from Table S1 is obtained from a line fit (pink) of the dotted black line between the two black circles.

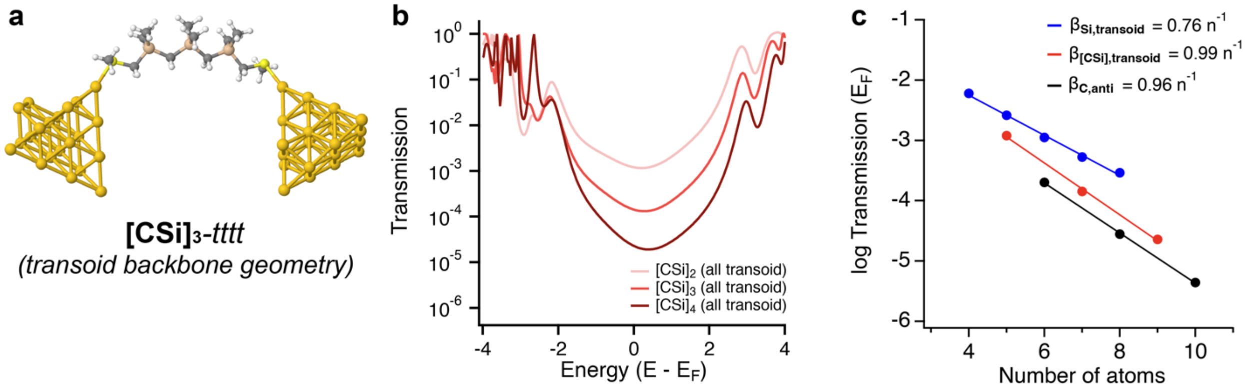

Supporting Figure S4. (a) DFT-optimized structure of a **[CSi]₃** junction, where the carbosilane backbone is in the all-*transoid* configuration. (b) Transmission functions plotted against energy (relative to the Fermi energy (E_F)) for the all-*transoid* conformers of **$[CSi]_{2\text{-}4}$** junctions. (c) The logarithm of transmission at the Fermi energy (*i.e.,* the zero energy point in (b)) plotted against the number of backbone atoms between the distal S atoms for the all-*transoid* conformers of **$Si_{2\text{-}6}$** and **$[CSi]_{2\text{-}4}$** and the all-*anti* conformers of **$C_{6,8,10}$**. We obtain the following transmission β values: β_{Si} = 0.76 n^{-1}, $\beta_{[CSi]}$ = 0.99 n^{-1}, β_C = 0.96 n^{-1}. Optimization and transmission calculation details can be found in the Calculations part of the Methods section in the manuscript.

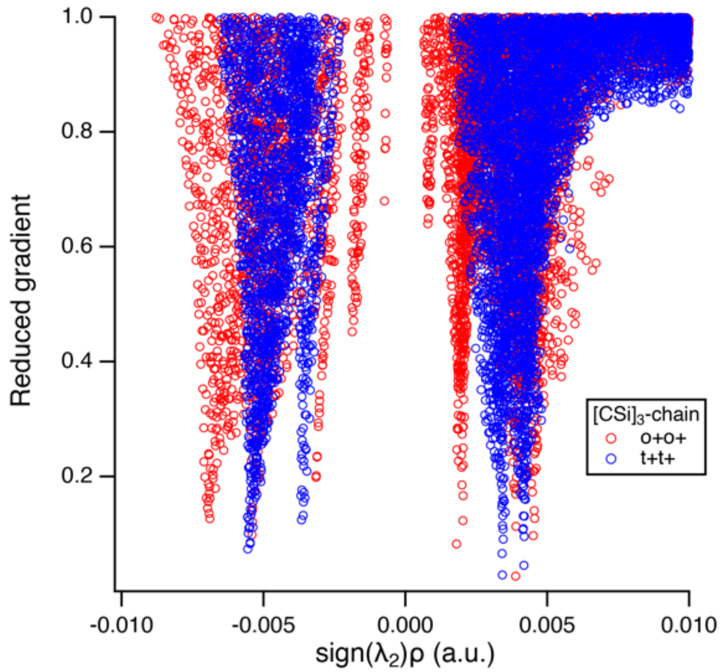

Supporting Figure S5. NCI comparisons via $s(\rho)$ plots for the SCF density of the o^+o^+ and t^+t^+conformers of **[CSi]$_3$-chain**. Favorable interactions appear on the left. The o^+o^+ conformer experiences stronger intramolecular non-covalent interactions, evinced by the greater density of negative (stabilizing) sign(λ_2)ρ species.

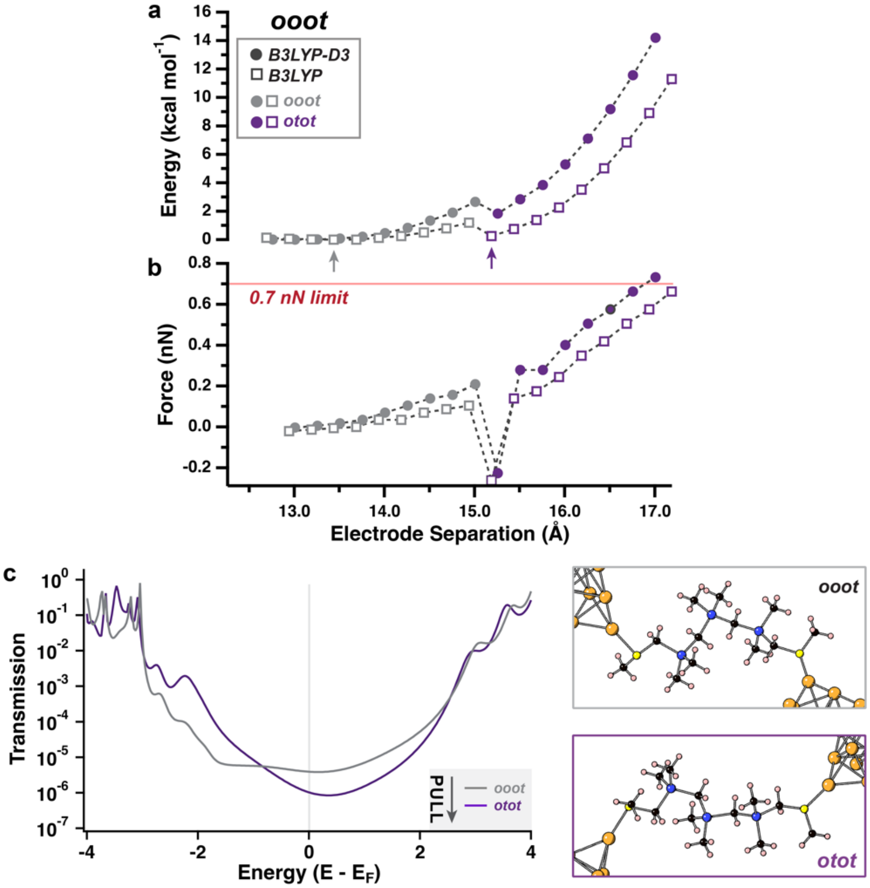

Supporting Figure S6. (a) Junction elongation plots track change in energy and backbone conformation as *ooot* **[CSi]**$_3$ initial geometries are stretched between Au_{10} pyramids until the junction breaks. Energies are plotted relative to the initial geometry optimized with either the B3LYP-D3 (filled circle) or B3LYP (empty square) functional. Color changes indicate a major change in backbone geometry. **(b)** Junction pulling force calculated from the change in energy with respect to electrode separation. Acute drops in force correspond to conformational changes to longer backbone dihedral geometries. **(c)** Transmission calculations and structures of Au_{20}-molecule-Au_{20} junctions with molecular geometries extracted from select points (marked by arrows in (a)) along the *ooot* pulling trajectory. The light gray vertical line is plotted at E_F as a visual aid. General note: all *ortho* (*o*) and *transoid* (*t*) conformers refer to o^+ or t^+ dihedral geometries.

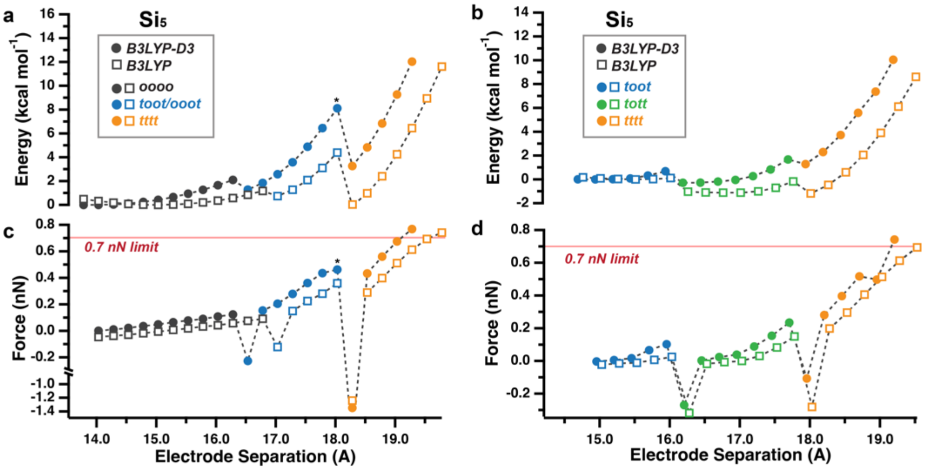

Supporting Figure S7. (a, b) Junction elongation plots that track the change in energy and backbone conformation as (a) *oooo* & (b) *toot* **Si$_5$** initial geometries are stretched between Au_{10} pyramids until the junction breaks. Energies are plotted relative to the initial geometry optimized with either the B3LYP-D3 (filled circle) or B3LYP (empty square) functional. Color changes indicate a major change in backbone geometry. The asterisk in (a) indicates a geometry change from *toot* to *ooot*. **(c, d)** Junction pulling force calculated from the change in energy with respect to electrode separation. Acute drops in force correspond to conformational changes to longer backbone dihedral geometries. We note that though *oooo & toot* is not a likely starting geometry for **Si$_5$** based on the DFT energies shown in **Figure 4b**, elongation simulations from this geometry serve to illustrate that fully coiled oligosilane geometries are readily stretched to fully *transoid* backbones in the permethyloligosilanes regardless of whether dispersion interactions are included in the calculation.

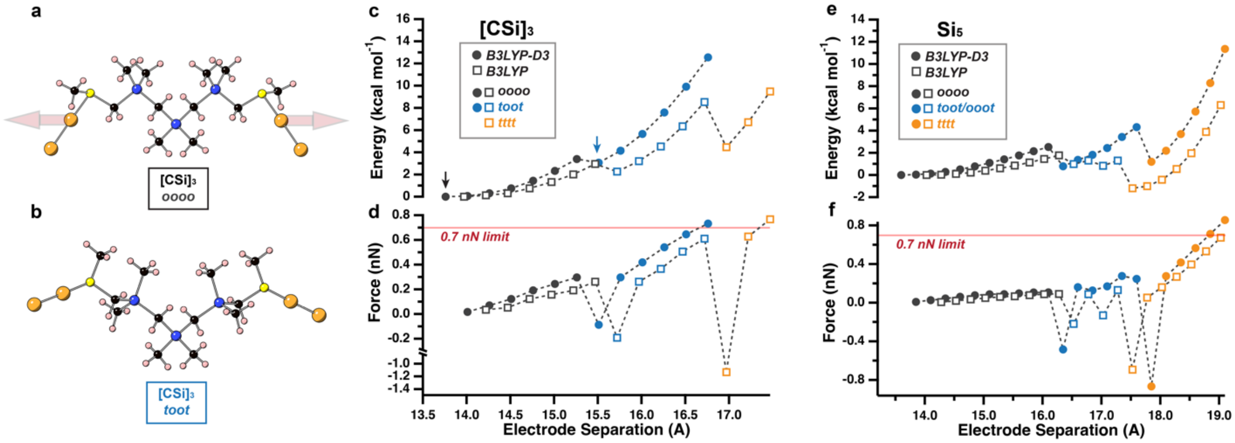

Supporting Figure S8. This figure indicates the same qualitative features in **Figure 5a,c** and **Figure S7a,c** can be recapitulated with simpler Au_2 diatom electrodes. **(a)** Structure of **[CSi]$_3$** appended to Au_2 diatoms optimized without Au-Au distance constraint from an initial *oooo* geometry at B3LYP-D3/6-31G(d,p) [light atoms], def2svp [Au atoms]. This is the starting structure from which the apex Au atoms (red arrows) are elongated in distance. This geometry is marked by the black arrow in (c). **(b)** Structure of the Au_2-**[CSi]$_3$**-Au_2 junction after the *oooo* geometry is pulled into a *toot* geometry (marked by the blue arrow in (c)). (**c**) Junction elongation plot that tracks the change in energy and backbone conformation as the *oooo*-disposed **[CSi]$_3$** is stretched between two Au_2 diatoms until the junction breaks (the 0.7 nN force limit of the dative thioether-Au contact is breached). Energies are plotted relative to the initial *oooo* geometry optimized with either the B3LYP-D3 (filled circle) or B3LYP (empty square) functional. **(d)** Junction pulling force

obtained from the change in energy with respect to electrode separation. **(e,f)** Energy and force of Au_2-**Si_5**-Au_2 junction stretching starting from initial *oooo* **Si_5** backbones. The all-*transoid* backbone geometry is accessed before the 0.7 nN limit is reached, regardless of whether the B3LYP or B3LYP-D3 functional is applied.

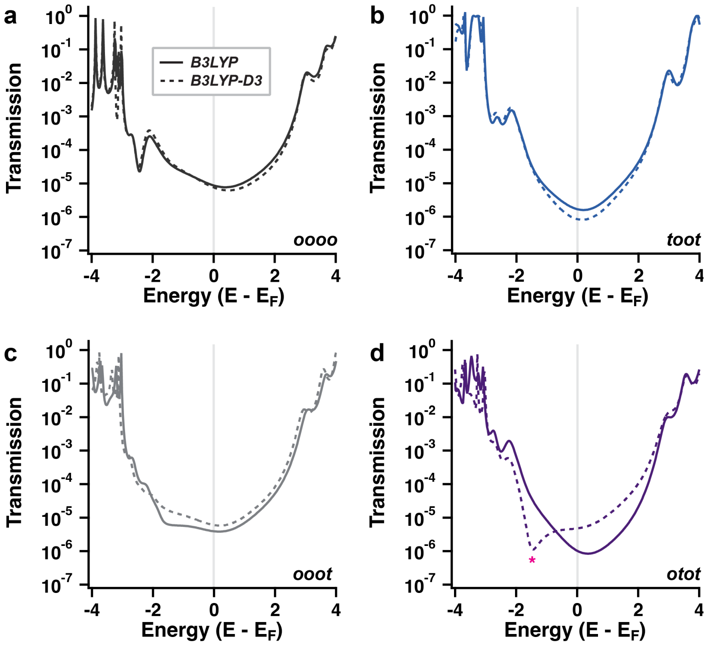

Supporting Figure S9. Transmission calculations of **$[CSi]_3$** geometries with (dotted line) and without (solid line) Grimme's D3 dispersion correction used to calculate the pulling trajectories in **Figure 5**. The light gray vertical line is a visual aid for evaluating transmission at the Fermi energy. We note that in some cases, as in *otot* (d)*,* dispersion corrections can cause new features such as a transmission anti-resonance (pink asterisk, -1.5 eV) indicative of destructive quantum interference.

Supporting Table S1. Conductance-displacement slopes obtained from line fitting of 2D histograms between junction startpoint and breakpoint.

	**β_{slope} / nm^{-1} [a]**
**C_6**	**2.12 ± 0.03**
**C_8**	**2.31 ± 0.02**
**C_{10}**	**2.81 ± 0.03**
**Si_2**	**3.11 ± 0.04**
**Si_3**	**0.26 ± 0.13**
**Si_4**	**0.11 ± 0.01**
**Si_5**	**1.11 ± 0.04**
**Si_6**	**0.00 ± 0.07**
**$[CSi]_2$**	**2.93 ± 0.10**
**$[CSi]_3$**	**4.01 ± 0.02**
**$[CSi]_4$**	**11.54 ± 0.05**
**solvent**	**21.31 ± 0.17**

Key. [a]Slopes obtained from line fits (pink lines in **Figure S2**) following the equation $G = G_c e^{-\beta L}$, where $\beta = \beta_{slope}$ and L = Au-Au displacement (nm).

Supporting Table S2. Dihedral geometries and free energies for **[CSi]₃-chain** conformations, sorted from longest to shortest $\Delta d_{Si(1)\text{-}Si(n)}$ length.

**[CSi]ₙ-chain**	**Dihedral geometry**[a] ($\omega_1\omega_2$)	$\Delta d_{Si(1)\text{-}Si(n)}$ (nm)[b]	ω_1[c]	ω_2[c]	ΔG^{298} (B3LYP-D3, kcal mol^{-1})[d]	ω_1[e]	ω_2[e]	ΔG^{298} (B3LYP, kcal mol^{-1})[d]
n=2	–	0.32	–	–	–	–	–	–
n=3	t^+t^+	0.65	163˚	163˚	0.00	165˚	165˚	0.00
	t^+t^-	0.65	166˚	–166˚	–0.38	168˚	–168˚	0.06
	t^+o^+	0.59	163˚	74˚	–0.44	164˚	74˚	–0.13
	t^+g^+	0.57	165˚	47˚	–0.63	164˚	50˚	0.13
	t^+o^-	0.57	162˚	–77˚	–0.63	168˚	–77˚	–0.13
	t^+g^-	0.57	171˚	–50˚	–0.56	165˚	–47˚	0.00
	o^+o^+	0.50	67˚	67˚	–1.51	74˚	74˚	–0.06

Key. [a] We define *gauche (g)* between 40-60˚, *ortho (o)* between 60-100˚, *transoid (t)* between 150-180˚. Only one sign of combinations are shown; the opposite signed conformers give identical Si-Si distances and energies. We exclude conformations that are saddle points or do not furnish local minima upon optimization at either the B3LYP/6-311G(d,p) or B3LYP-D3/6-311G(d,p) level. [b]Through-space distance between Si_1 and Si_2 centers for the **[CSi]₂-chain** or Si_1 and Si_3 centers for the **[CSi]₃-chain** calculated at the B3LYP-D3/6-311G(d,p) level of theory. [c] C-Si-C-Si dihedral angles calculated at the B3LYP-D3/6-311G(d,p) level. [d] Free energies relative to the t^+t^+ conformer calculated with the listed functional with the 6-311G(d,p) basis set. [e] C-Si-C-Si dihedral angles calculated at the B3LYP/6-311G(d,p) level.

[CSi]₄-chain

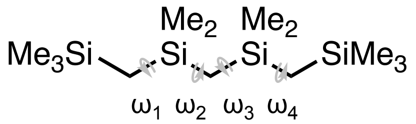

Supporting Table S3. Geometric parameters and free energies for selected **[CSi]₄-chain** conformers, sorted from longest to shortest $\Delta d_{Si(1)\text{-}Si(4)}$ length.

**Dihedral geometry**[a] ($\omega_1\omega_2\omega_3\omega_4$)	$\Delta d_{Si(1)\text{-}Si(4)}$ (nm)[b]	ΔG^{298} (B3LYP-D3, kcal mol^{-1})[c]	ΔG^{298} (B3LYP, kcal mol^{-1})[c]	ω_1[d]	ω_2[d]	ω_3[d]	ω_4[d]
$t^-t^-t^-t^-$	0.97	0.00	0.00	–163˚	–163˚	–163˚	–163˚
$t^-o^+t^-t^-$	0.87	–0.44	0.31	–162˚	77˚	–162˚	–163˚
$o^+t^-t^-t^-$	0.85	–0.56	0.00	77˚	–163˚	–165˚	–162˚
$t^-o^+t^-g^-$	0.84	–1.00	–0.19	–162˚	78˚	–164˚	–47˚
$o^+o^+o^+t^-$	0.79	–1.76	0.25	66˚	69˚	77˚	–164˚
$o^+o^+o^+o^+$	0.78	–1.51	–0.06	70˚	64˚	64˚	70˚
$o^+o^+t^-t^-$	0.76	–1.38	0.13	65˚	67˚	–162˚	–162˚
$t^-o^+o^+t^-$	0.76	–1.32	0.25	–160˚	78˚	78˚	–160˚
$t^-o^+t^-o^+$	0.74	–1.07	–0.19	–162˚	76˚	–161˚	77˚
$o^+t^-t^-o^-$	0.70	–1.00	0.06	77˚	–160˚	–167˚	–74˚
$\boldsymbol{o^+o^+t^-g^-}$	**0.69**	**–2.38**	**0.06**	**62˚**	**69˚**	**–160˚**	**–45˚**

$g^-t^-t^-g^-$	0.64	–1.69	–0.06	–43˚	–163˚	–163˚	–43˚
$t^-o^+g^-t^-$	0.63	–1.76	–0.19	–158˚	79˚	–45˚	–170˚
$o^+o^+t^-o^+$	0.62	–1.76	0.19	70˚	62˚	–175˚	63˚
$o^+o^+g^-t^-$	0.58	–2.07	0.00	66˚	61˚	–50˚	–160˚

Key. [a] We define *gauche (g)* between 40-60˚, *ortho (o)* between 60-100˚, *transoid (t)* between 150-180˚. [b] Through-space distance between Si_1 and Si_4 centers, $\Delta\mathbf{d}_{Si(1)-Si(4)}$, calculated at the B3LYP-D3/6-311G(d,p) level of theory. [c] Free energies relative to the $t^-t^-t^-t^-$ conformer calculated with the listed functional with the 6-311G(d,p) basis set.. [d] Dihedral angles from calculations at the B3LYP-D3/6-311G(d,p) level of theory.

Supporting Table S4. Energies of free molecules, Au_{10}-bound molecules, and binding energy per dative $Au-SR_2$ bond found from DFT structure optimizations.

	$\mathbf{E}_{mol}$ / *hartree* [b]	$\mathbf{E}_{junction}$ / (*hartree*) [c]	$\Delta\mathbf{E}_{per\ bond}$ / *kcal mol*$^{-1}$ (*eV*)[d]
**C₄**[a]	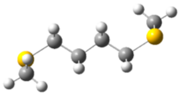 -1033.489217	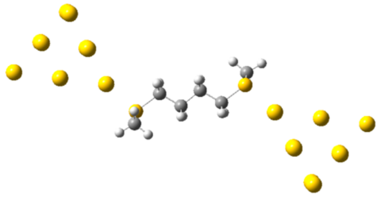 -3749.76097	14.7 (0.64)
**[CSi]₃**, *oooo*	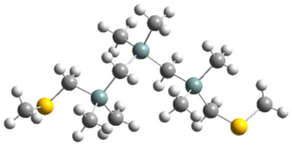 -2141.6274	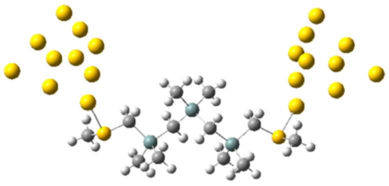 -4857. 897793	14.3 (0.62)
**[CSi]₃**, *ooot*	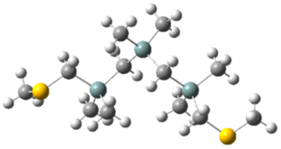 -2141.6263	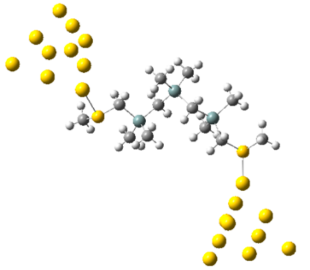 -4857.8977	14.7 (0.64)
**[CSi]₃**, *toot*	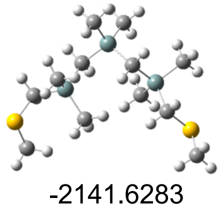 -2141.6283	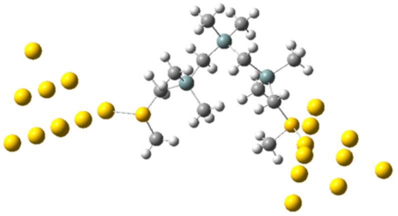 -4857.897021	13.8 (0.60)

Key. [a]**C_4** was selected for comparison with **$[CSi]_3$**, as its junction breaking force was evaluated in Ref. 1. [b]Molecules optimized with terminal Me-S-C-X dihedrals in *ortho* (90˚) starting geometries at B3LYP-D3/6-31G(d,p) level of theory. [c] Au_{10} pyramids were appended to each S atom with starting Au-S-C bond angles and lengths of 110˚ and 2.48 Å, then optimized at B3LYP-D3/6-31G(d,p)/def2-SVP(Au) level of theory. [d]Binding energy (ΔE) per $Au-SR_2$ bond calculated from the

formula: $\Delta E = [(\mathbf{E_{mol}} + 2\mathbf{E_{Au10}}) - \mathbf{E_{junction}}] / 2$. $\mathbf{E_{Au10}}$ = -1358.1124 Hartree, which was obtained from single-point calculations at the B3LYP-D3/def2-SVP level of theory.

Supporting Note 1

It is important to also consider the entropic implications of a σ-bonded chain with sterically equivalent rotational states.[2] Even in the absence of dispersion interactions, there are many kinked conformational states that populate the conformational equilibrium than the all-*transoid* state. This means there is a greater probability that a partially coiled conformer for the carbosilanes is picked up in the junction in the first place, compared to the mostly *anti* alkane or *transoid* oligosilane wires. And yet, entropic effects alone do not account for the experimental step length trends. If there were no significant dispersion interactions in **[CSi]$_3$-chain** and all conformers were isoenergetic, one would expect an average Si_1-to-Si_3 distance of 0.59 nm, *i.e.*, a length extension of 0.26 nm from **[CSi]$_2$-chain** (**Table S2**). But our experimental data suggests this length extension should be significantly smaller: adding a C-C bond to the alkane series results a 0.25 nm length increase, yet our step length data in **Table 1** indicates that the Δz for the **[CSi]$_n$** series is significantly shorter than that of the **C$_n$** series. This analysis supports that the short step lengths we observe do not merely occur from entropic effects, but depend heavily on strong intramolecular dispersion interactions that bias conformation toward short, kinked junction conformers.

A similar interpretation can be extended to **[CSi]$_4$-chain**. There are more geometries to consider for **[CSi]$_4$-chain**, with four variable dihedral positions. The list of conformers in **Table S3** is not meant to be comprehensive, but to provide a general sense for how Si_1-Si_4 distances and free energies vary as *g* and *o* geometries are introduced, with and without dispersion corrections. We chose this set of conformers from initial combinations of either 180˚, 60˚, or –60˚ for ω_1, ω_2, ω_3, and ω_4 as the barrier between *t*+ and *t*- geometries and *g*± and *o*± geometries are low. Starting geometries with significant *syn*-pentane or related intramolecular repulsions were excluded. **Table S3** shows that in the absence of dispersion corrections, each stable dihedral configuration is within 0.3 kcal mol^{-1} of the fully transoid geometry. With dispersion corrections, we find that the fully transoid conformer is 2.4 kcal/mol higher in energy than the lowest lying $g^+g^+t^+g^+$ kinked state. We note that for this conformer $d_{Si1\text{-}Si4}$ = 6.9 Å, significantly shorter than the fully *transoid* length, yet 1.9 Å longer than the most stable conformer of **[CSi]$_3$-chain**.

Supporting Note 2

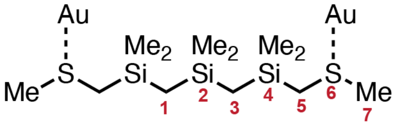

The structural simplicity and chemically equivalent endgroups of **[CSi]$_3$-chain** allowed us to focus on only two equivalent C_1-Si_2-C_3-Si_4 internal backbone dihedrals (**D1**). However, the conformational landscape for **[CSi]$_3$** is far more complex than for **[CSi]$_3$-chain**, with six additional electronically relevant and geometrically distinct dihedral angles to consider: **D2** Si_2-C_3-Si_4-C_5 (t^+, t^-, g^+, g^-, o^+, o^-), **D3** C_3-Si_4-C_5-S_6 (*a*, g^+, g^-), **D4** Si_4-C_5-S_6-C_7 (*a*, o^+, o^-), on each side of the molecule. A comprehensive investigation of each eight-dihedral permutation and its junction stretching trajectory and transmission is computationally expensive and outside the scope of this manuscript, which is to explore how intramolecular dispersion in dimethylsilmethylene oligomers may preclude all-*transoid* geometries from being appreciably accessed in carbosilane junctions.

We can achieve a broad-strokes understanding of this by modeling a focused set of **[CSi]$_3$** conformers. Building off the finding in **Figure 4** that o^+o^+ internal kinks are the most energetically stabilized by dispersion, we focus our investigation on what happens as these internal *ortho* kinks are pulled apart in the presence or absence of dispersion interactions. Adjacent o^+o^- are sterically

disfavored while the energy differences between o^+/g^+ and t^+/t^- configurations are trivial: this leads us to explore $o^+o^+o^+o^+$, $o^+o^+o^+t^+$, and $t^+o^+o^+t^+$ as representative (but incomprehensive) configurations of the four innermost backbone dihedrals. Meanwhile, we choose **D3** = 180˚ (*anti*) and **D4** = 90˚ (*ortho*) dihedrals on each side of the molecule as initial geometries, as these geometries are *anti* to the gold electrodes and are thus colinear with the axis of pulling from each electrode.

II. Synthetic Procedures and Characterization of Compounds

1. General Synthesis and Characterization Information

All reactions were performed in oven-dried glassware equipped with Teflon magnetic stir bars and conducted in a nitrogen atmosphere using a Schlenk manifold. All reaction solvents were degassed with argon and dried with activated alumina columns through a solvent purification system (JC Meyer) and stored over 4 Å molecular sieves. Silica plugs were conducted using 230-400 mesh silica (SiliaFlash P60, Silicycle).

Materials. All chemicals were purchased from commercial sources and used without further purification. Dichlorodimethylsilane, N,N,N′,N′-Tetramethylethylenediamine, dimethyl sulfide and 2.5 M n-butyllithium in hexanes were purchased from Sigma Aldrich. Tetrahydrofuran and pentane for use in solvent purification systems were purchased from Fisher. Magnesium turnings were purchased from Strem. Chloro(chloromethyl)dimethylsilane was purchased from TCI Chemicals.

Instrumentation. ^{1}H NMR, ^{13}C NMR and ^{29}Si NMR were recorded on a Bruker AV 600 (600 MHz) spectrometer. Chemical shifts for ^{1}H NMR are reported in units of parts per million downfield from tetramethylsilane and referenced to residual protium in NMR solvents ($CHCl_3$, δ 7.26). Chemical shifts for 13C NMR are reported in parts per million downfield from tetramethylsilane and are referenced to the center peaks of residual solvents (CHCl3, δ 77.16). Chemical shifts for silicon are reported in parts per million downfield from tetramethylsilane and are referenced to a tetramethylsilane internal standard, or in the case of **[CSi]$_4$**, an external tetramethylsilane standard. To compensate for the low isotopic abundance of 29Si, the DEPT pulse sequence was employed for the amplification of the signal. Data are represented as follows: chemical shift, multiplicity (s = singlet, d = doublet, t = triplet, m = multiplet, br = broad), coupling constants in Hertz, and integration. High-resolution mass spectrometry (HRMS) was recorded on a Waters XEVO G2XS QToF mass spectrometer equipped with a UPC2 SFC inlet, electrospray ionization (ESI) probe, atmospheric pressure chemical ionization (APCI) probe, and atmospheric solids analysis probe (ASAP+).

2. NMR Spectra

[CSi]₂ ^{1}H NMR (600 MHz, $CDCl_3$)

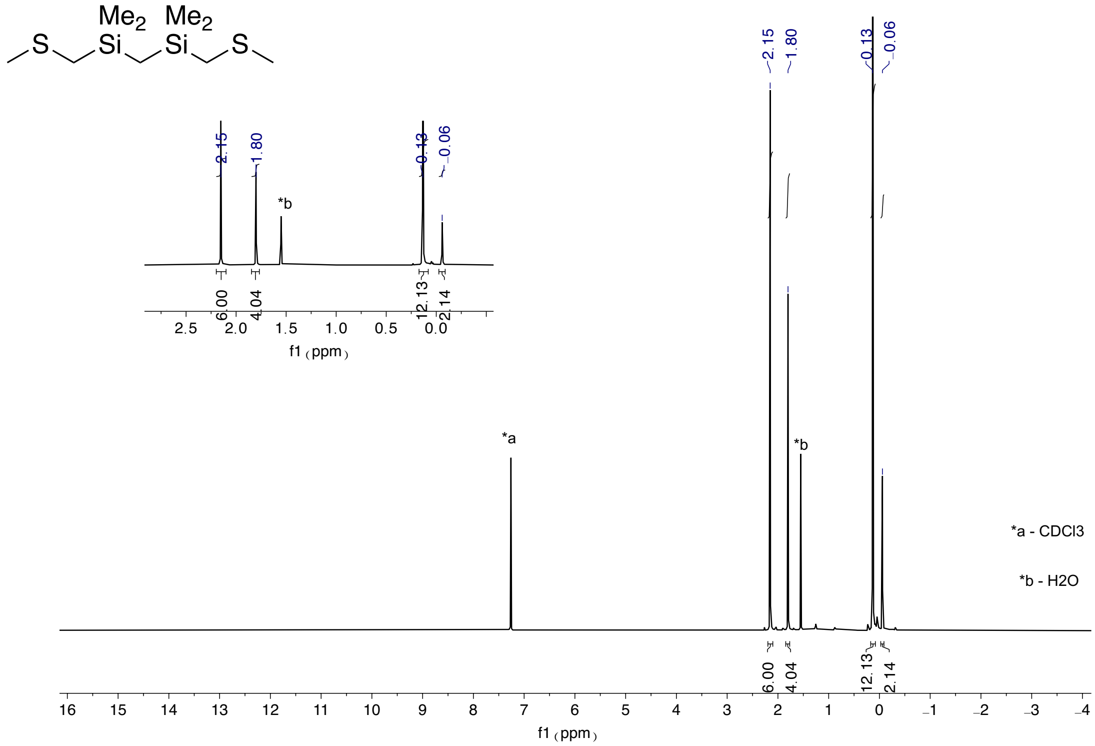

[CSi]₂ ^{13}C NMR (151 MHz, $CDCl_3$)

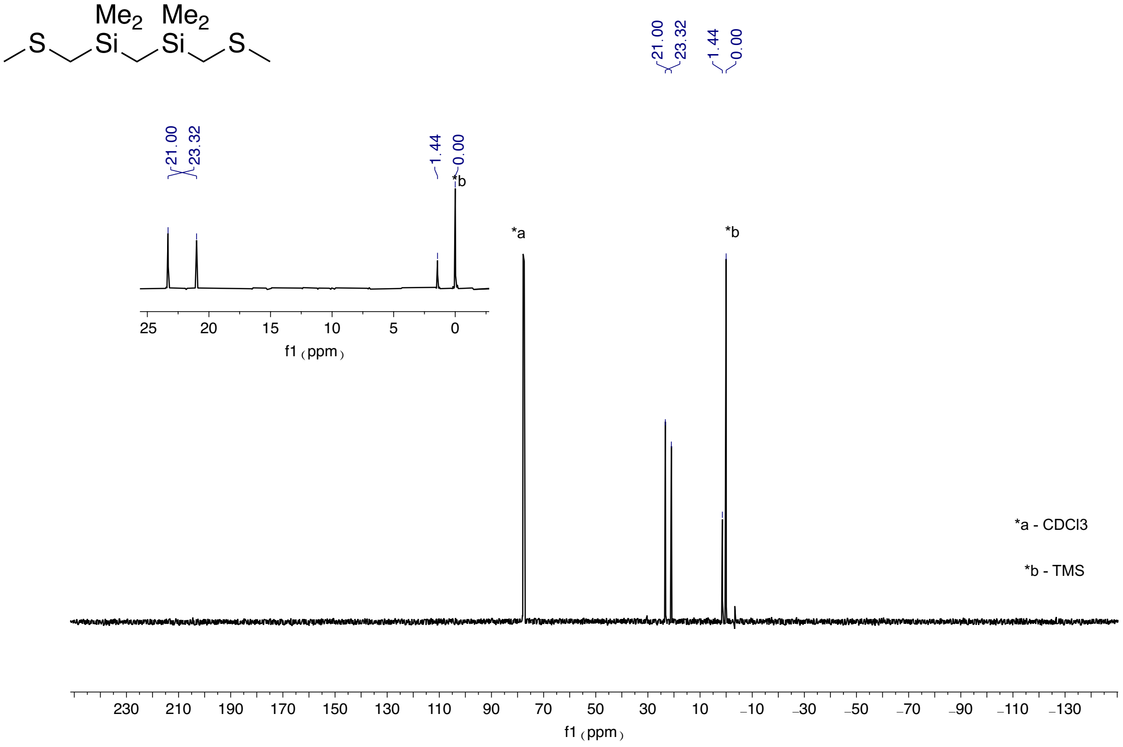

[CSi]₂ ^{29}Si NMR (79 MHz, $CDCl_3$)

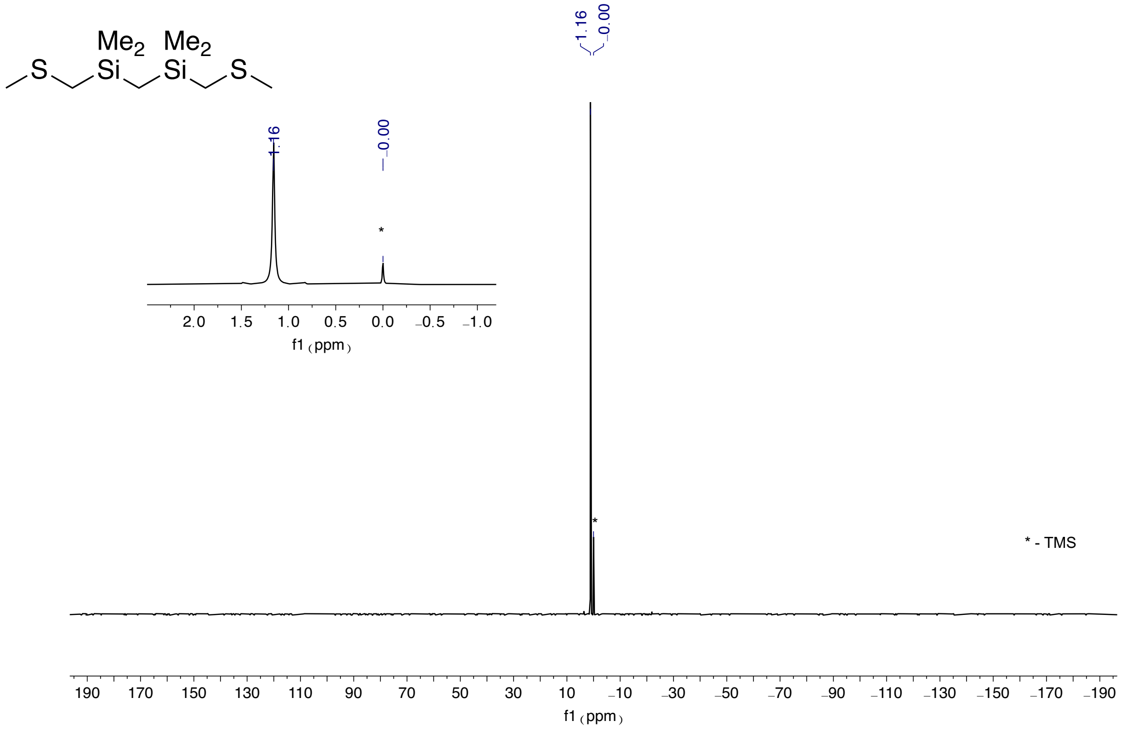

[CSi]₃ ^{1}H NMR (600 MHz, $CDCl_3$)

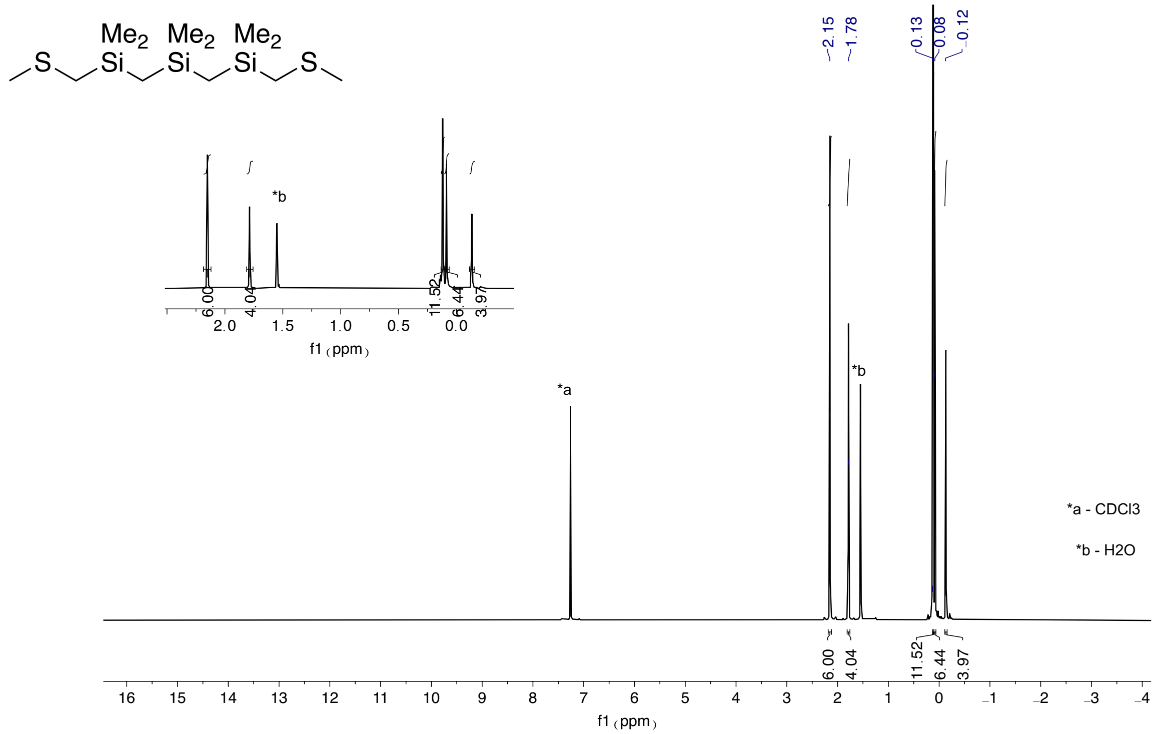

[CSi]$_3$ ^{13}C NMR (151 MHz, $CDCl_3$)

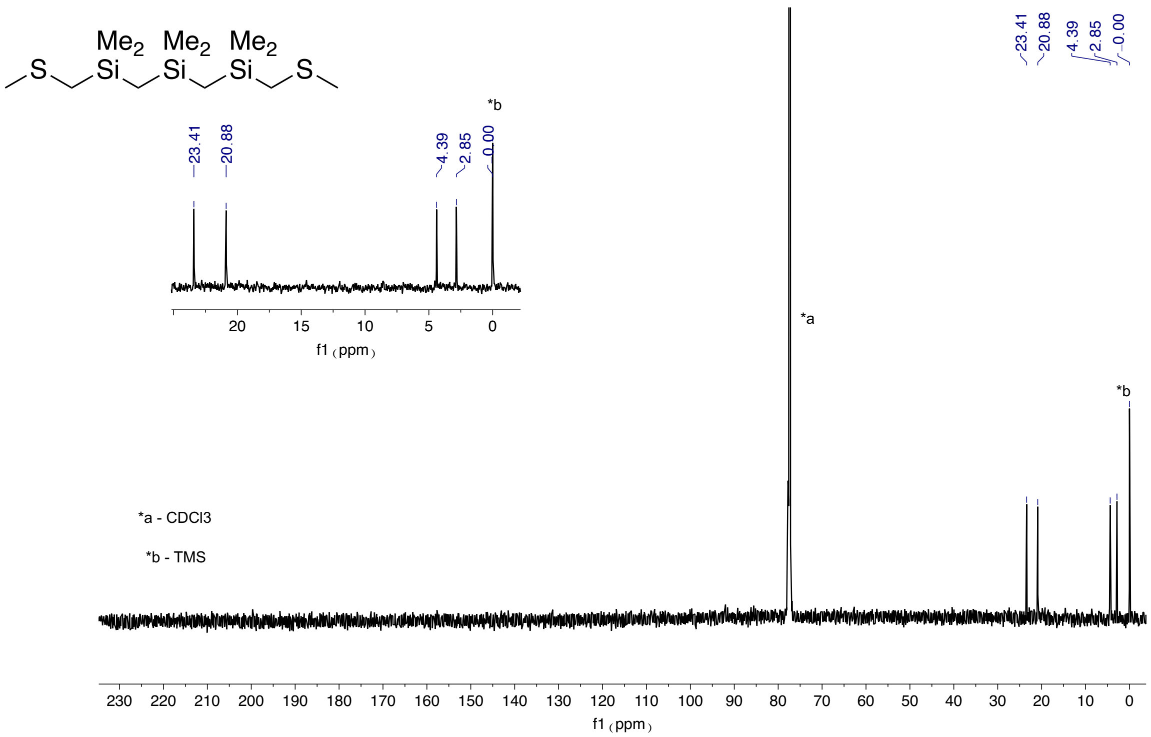

[CSi]$_3$ ^{29}Si NMR (79 MHz, $CDCl_3$)

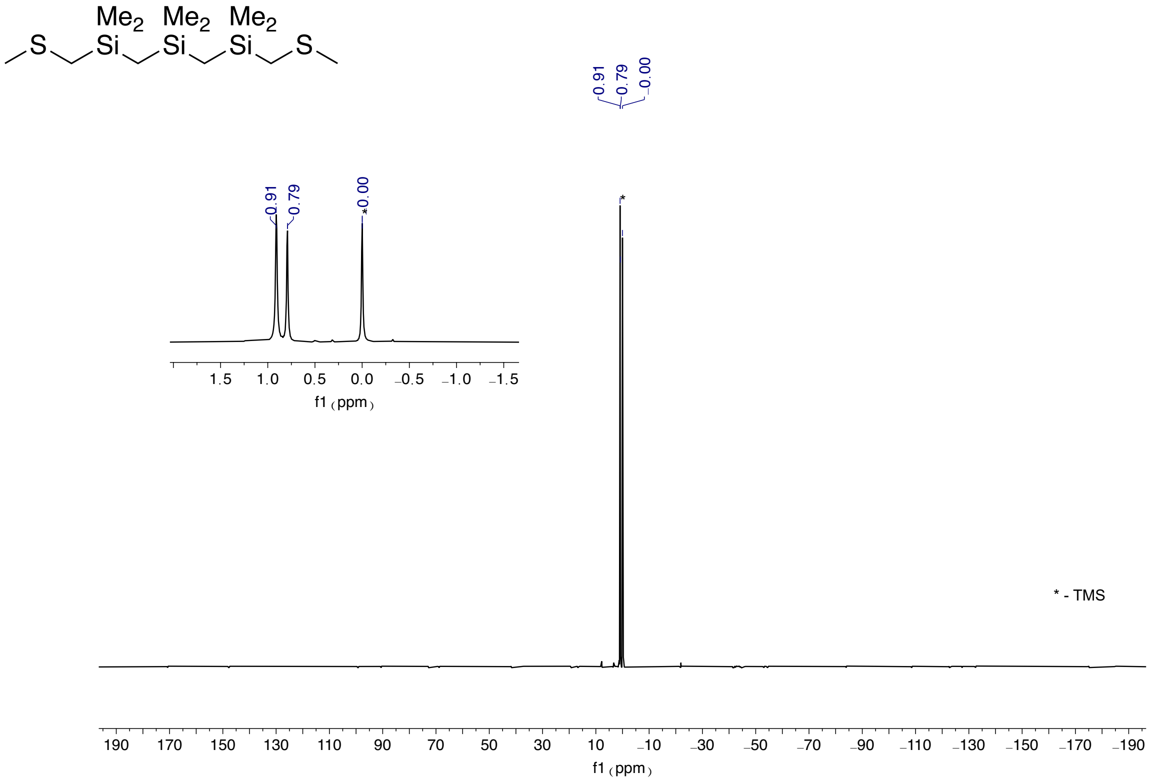

[CSi]$_4$ ^1H NMR (600 MHz, $CDCl_3$)

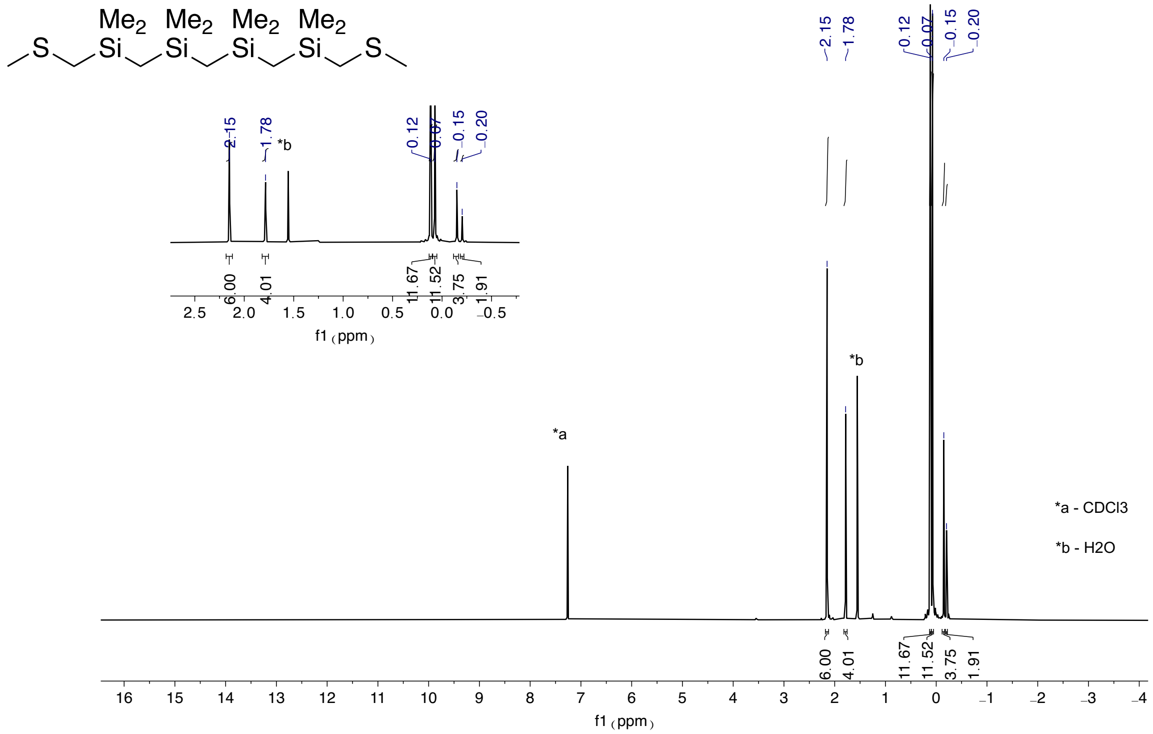

[CSi]$_4$ ^{13}C NMR (151 MHz, $CDCl_3$)

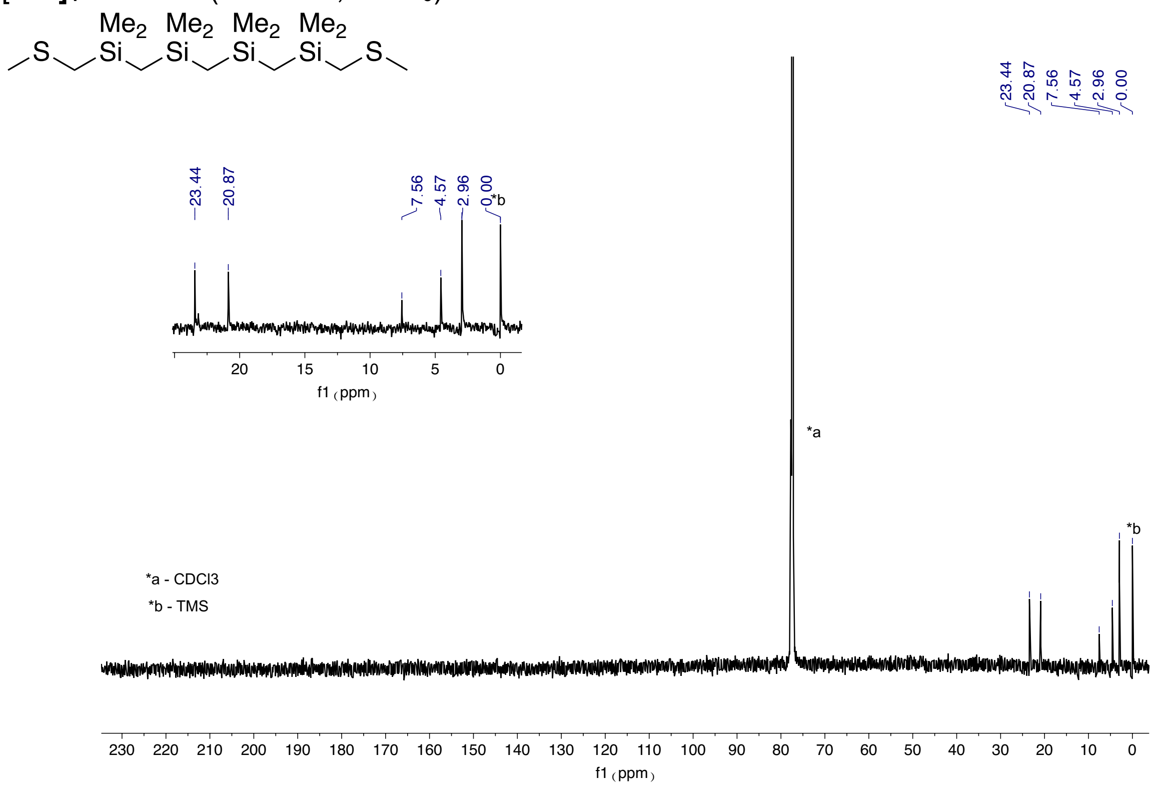

[CSi]$_4$ ^{29}Si NMR (79 MHz, $CDCl_3$)

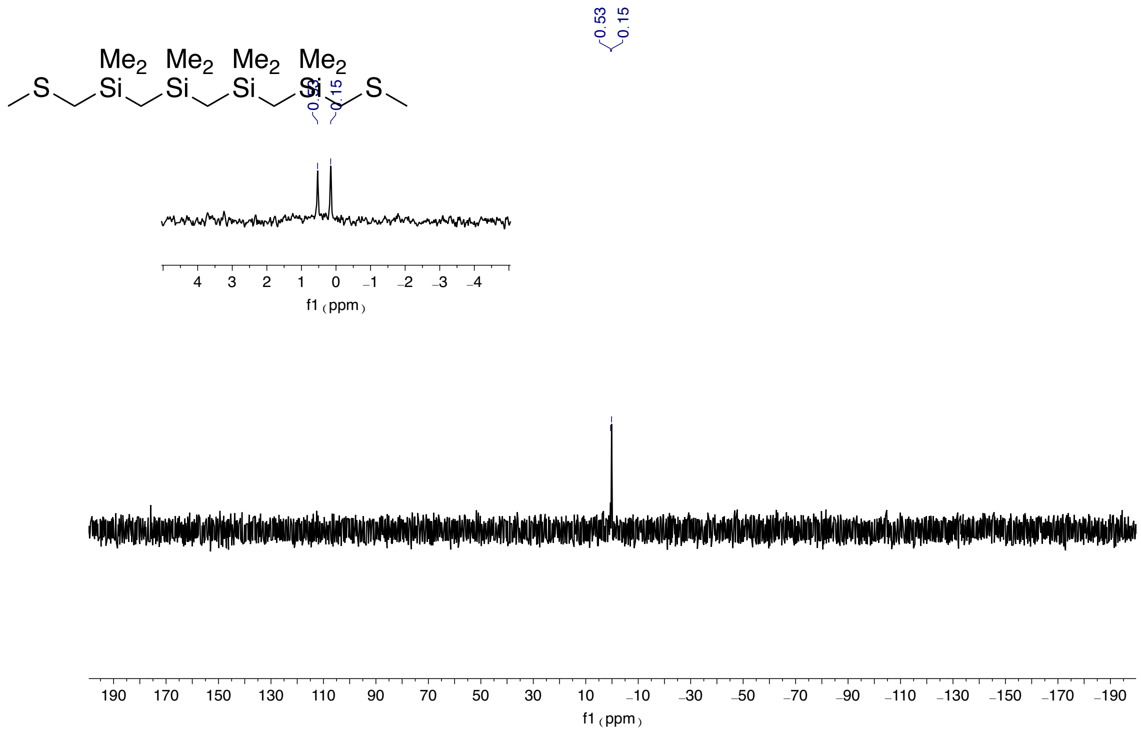

III. References

(1) Frei, M.; Aradhya, S. V.; Hybertsen, M. S.; Venkataraman, L. Linker Dependent Bond Rupture Force Measurements in Single-Molecule Junctions. *J. Am. Chem. Soc.* **2012**, *134* (9), 4003–4006. https://doi.org/10.1021/ja211590d.

(2) Flory, P. J. Foundations of Rotational Isomeric State Theory and General Methods for Generating Configurational Averages. *Macromolecules* **1974**, *7* (3), 381–392. https://doi.org/10.1021/ma60039a022.
